# Shifts in the Rhizosphere Bacterial Community and Improved Essential Oil Yield and Quality in *Chamomilla recutita* L. Plant Through Cyanobacterial Inoculation

**DOI:** 10.1007/s00248-026-02815-1

**Published:** 2026-06-29

**Authors:** Doaa Ibrahim, Afaf H. Ali, Ahmed Refaat, Rehab Z. Abdallah, Sameh AbouZid, Tarek Elsayed, Mehrshan El Mokadem, Gabriele Berg, Eman Nour

**Affiliations:** 1 Faculty of Organic Agriculture, Heliopolis University, Cairo, Egypt; 2 Botany Department, Women’s College, Ain Shams University, Cairo, Egypt; 3 Research and Development Department, Faculty of Pharmacy, Heliopolis University, Cairo, Egypt; 4 Department of Bioinformatics and Genomics, College of Biotechnology, Misr University for Science and Technology, Giza, Egypt; 5 Biology department, School of Sciences and Engineering, The American University in Cairo, Cairo, Egypt; 6 Department of Pharmacognosy, Faculty of Pharmacy, Beni-Suef University, Beni-Suef, 62514 Egypt; 7 Microbiology Department, Faculty of Agriculture, Cairo University, Cairo, Egypt; 8 Institute for Environmental Biotechnology, Graz University of Technology, Graz, Austria; 9 Leibniz Institute for Agricultural Engineering and Bioeconomy (ATB), Potsdam, Germany; 10 Institute for Biochemistry and Biology, University of Potsdam, Potsdam, Germany

**Keywords:** Cyanobacteria, Biofertilizer, Essential oil, Apigenin, Rhizosphere bacterial community

## Abstract

Rhizosphere bacterial communities play a crucial role in improving nutrient availability, disease and stress resistance, and overall development in plants. Biofertilizers can enhance plant growth, but little is known about their mode of interaction. Here, we studied the impact of seedling coating with two nitrogen-fixing cyanobacterial strains, *Nostoc* sp. NoHu, or *Anabaenopsis circularis* AnHu, on chamomile (*Chamomilla recutita* L.) growth, essential oil yield and quality, apigenin-7-O-glucoside, and the composition of rhizosphere bacterial communities, under field conditions in Egypt. The rhizosphere bacterial community, analyzed by 16 S rRNA amplicon libraries, composed of 31 phyla and 164 different genera. Compared to untreated plants, the application of both strains showed contrasting effects. *Nostoc* sp. application significantly enhanced almost all tested plant growth parameters, including fresh and dry weights of shoots, roots, and flowers, as well as the yield and content of vital essential oil constituents and the apigenin-7-O-glucoside. Furthermore, *Nostoc* sp. application increased both the abundance and diversity of the rhizosphere bacterial community compared to untreated plants, suggesting an indirect effect mediated through altering the rhizosphere bacterial community structure. In contrast, *A. circularis* application negatively affected essential oil yield and quality, exhibited the lowest bacterial abundance and diversity. These findings highlight potential of *Nostoc* sp. NoHu to boost chamomile productivity and essential oil quality. The contradictory responses between the two cyanobacterial strains emphasize strain-specific effects with *Nostoc* sp. NoHu as a promising biofertilizer candidate for chamomile.

**Supplementary Information:**

The online version contains supplementary material available at 10.1007/s00248-026-02815-1.

## Introduction

Plants and their associated microorganisms form functional assemblages that are referred to as plant holobionts [1, 2]. The plant microbiota is mainly assembled from seed and soil microorganisms and forms distinct microbial communities in each plant compartment [3, 4]. The soil-root interface, also known as the rhizosphere, is a hotspot for plant-microbe interactions with increased abundance and activity of some bacterial population proliferating in response to the root exudate mixture [5, 6]. Specific plant-microbe networks have likely emerged from co-evolution [7], and were shaped by domestication and breeding [3, 8]. Moreover, intense agriculture, especially chemical pesticides and fertilizers, are strong drivers of the plant microbiome. This shift is characterized by a decrease of diversity, symbionts and host specificity, and an increase of r-strategic microbes, pathogens, and hypermutators [9]. In long term, this has consequences for plant and planetary health and needs action.

Microbiome-based biofertilizers, -stimulants and -pesticides provide an environmentally friendly alternative to chemical treatments [10]. For their efficient application and consistent effects, their mode of interaction has to be fully understood. While their effect on plants and pathogens is well studied, the interaction with the plant microbiota was only recently brought in focus [11]. Today, mainly spore-forming bacteria are developed into commercial products. Cyanobacteria, a diverse group of photosynthetic microorganisms, are an interesting alternative because they are vital components of the soil and rhizosphere microbiota with remarkable beneficial properties, including N_2_ and CO_2_ fixation abilities and cycling of different essential nutrients. In addition, they are known for their production of various plant growth promoting substances such as, auxins, gibberellins, cytokinin, brassinosteroid, and abscisic acid [12, 13]. The beneficial impact of cyanobacteria-based biofertilizers stimulating plant growth and productivity, has been reported for various crops, including rice, wheat, tomatoes, maize, peas, and cotton [14]. Although the efficiency of cyanobacteria as biofertilizers for various crops are well-documented, their impact on the bioactive secondary metabolite content and quality of medicinal plants is less explored [15, 16]. Beyond their direct contributions to nutrient cycling and plant growth regulation, cyanobacteria also have the capacity to shape the rhizosphere microbial community in a manner that promote the plant growth [17]. Cyanobacteria were shown to modulate the composition and diversity of microbial community, fostering beneficial microbial interactions as an indirect strategy for improving plant growth [17]. This ability to modulate the rhizosphere environment highlights the potential of cyanobacteria to act as biofertilizers that go beyond nutrient supplementation. Despite that, not much attention has been paid to their impact on the rhizosphere microbial community, particularly, in medicinal plants [18]. Therefore, we aimed to study how cyanobacteria can shift the rhizosphere bacterial community of medicinal plants and the subsequent impact on the growth performance and production of bioactive compounds.

Chamomile (*Matricaria chamomilla* L.) is a widely cultivated medicinal plant known for the pharmaceutical properties of its essential oils and bioactive compounds. The most useful constituents of the essential oil of chamomile are chamazulene and α-bisabolol [19] in addition to apigenin, a flavonoid compound found in chamomile flowers [20]. These compounds possess valuable therapeutic properties, including anti-inflammatory, antioxidant, and anti-carcinogenic effects [21]. While the demand for high-quality chamomile products has increased, traditional agricultural practices, however, often face challenges in optimizing the production of these valuable bioactive compounds. As rhizosphere microbiota can influence the accumulation of bioactive compounds in medicinal plants [22], understanding the relationship between the rhizosphere microbiome and the accumulation of bioactive compounds is needed to develop proper bioproducts. The use of cyanobacteria as biofertilizers is gaining more attention as a sustainable agricultural practice, offering an environmentally friendly alternative to chemical fertilizers.

Present study aimed to investigate the effects of inoculating two nitrogen-fixing cyanobacterial strains, *Nostoc* sp. NoHu and *Anabaenopsis circularis* AnHu, on the rhizosphere bacterial community structure and the production of bioactive secondary metabolites in field-grown chamomile plants (*Chamomilla recutita* L.). Selected cyanobacterial strains were obtained from biodynamically managed soils at Sekem farms in Egypt, based on their biological nitrogen fixation abilities [23]. We hypothesized that, in addition to the known roles in the biological nitrogen fixation and the production of phytohormones and other bioactive metabolites, the beneficial effects of cyanobacteria may also be mediated by their ability to modulate the rhizosphere bacterial community composition. To address this, we employed a polyphasic approach combining (i) cultivation-based methods for the determination of colony-forming units, (ii) high-throughput 16 S rRNA gene sequencing for a barcoding bacterial communities, (iii) assessment of plant traits to evaluate growth performance, and (iv) chemical analytics (GC-MS and HPLC) for determining essential oil yield and quality, as well as apigenin-7-O-glucoside content according to the 29th edition of the United States Pharmacopeia (USP).

## Materials and Methods

### Experimental Site Description

A field experiment was carried out at the experimental area of Heliopolis University for Sustainable Development, Egypt. The experimental site was arranged in a randomized complete block design with three treatments and three replicates. Each block contained all three treatments, which were randomly assigned to plots within the block. Each plot comprised three rows, with nine plants per row, providing adequate representation of plant growth under field conditions. To minimize the effects of soil heterogeneity, the field was divided into blocks according to spatial variation across the site. Prior to the experiment, composite soil samples were collected from a depth of 0–15 cm and subjected to physicochemical analysis. The soil was characterized by a pH of 8.85, 1.8 dS/m electrical conductivity, 1.02% organic matter, 0.59% organic carbon, 186.667 mg/kg available nitrogen, 31 mg/kg available phosphate, and 493 mg/kg available potassium.

### Cyanobacterial Inoculum Preparation

Two nitrogen-fixing cyanobacteria strains, *Nostoc* sp. NoHu and *A*. *circularis* AnHU [23], were enriched in BG-11_0_ broth medium [24], separately. After three-four weeks, cells were harvested by centrifugation at 14,000 x *g* (High-speed centrifuge, A&E lab., U.K.) for 15 min and the resulted pellets were resuspended in sterilized tap water. The cell density was adjusted to 0.2% cyanobacteria cell suspension (2.0 g fresh cells dissolved in 1000 ml sterilized tap water) [25].

### Experimental Design

Chamomile (*Chamomilla recutita* L.) seedlings, obtained from El-Mizan plant raising company, were treated via root-dipping in suspensions of *Nostoc* sp. NoHu or *A. circularis* AnHu strains, prepared as mentioned above. Chamomile seedlings dipped in sterilized tap water were used as untreated plants. Nine plant replicates per plot were used for each treatment. Twenty days after planting, 100 ml of each corresponding cyanobacteria suspension (0.2%) was applied to the soil around the roots, followed by post-inoculation every 20–25 days until the end of the experiment. 100 ml of tap water was applied for untreated plants correspondingly. Forty days after transplanting, the chamomile plants came into flower. Flowers were harvested every 7–10 days, counted and weighted freshly for each plant and then stored at −20 ℃ until the end of the experiment for further analysis.

### Samples Collection and Plant Growth Parameters Assessment

Five months after planting, plants of each treatment were separately collected. Plant growth related parameters including, root and shoot fresh and dry weight, after drying at 60℃ for 72 h until the weight remained constant, were determined. Shoot to root ratio was calculated using the dry weights. In addition, the flowers fresh weight of each plant was determined at the end of the experiment.

### Extraction and Chemical Analysis of Chamomile Essential Oil

Essential oil was obtained from 50 g sample of dried flowers collected from each treatment in 500 ml of distilled water, using Clevenger’s apparatus [25]. The extraction was continued for 5 h from the start of condensation, then extracts were dried over anhydrous Na_2_SO_4_. The yield of the essential oil was calculated for each treatment according to the equation [26]:

$$\:essential\:oil\:yield=\:\frac{\mathrm{M}\mathrm{a}\mathrm{s}\mathrm{s}\:\mathrm{o}\mathrm{f}\:\mathrm{e}\mathrm{s}\mathrm{s}\mathrm{e}\mathrm{n}\mathrm{t}\mathrm{i}\mathrm{a}\mathrm{l}\:\mathrm{o}\mathrm{i}\mathrm{l}\:\mathrm{o}\mathrm{b}\mathrm{t}\mathrm{a}\mathrm{i}\mathrm{n}\mathrm{e}\mathrm{d}}{\mathrm{F}\mathrm{l}\mathrm{o}\mathrm{w}\mathrm{e}\mathrm{r}\mathrm{s}\:\mathrm{d}\mathrm{r}\mathrm{y}\:\mathrm{w}\mathrm{e}\mathrm{i}\mathrm{g}\mathrm{h}\mathrm{t}}\times\:100$$

Gas chromatography (GC) analysis of chamomile-extracted essential oil was performed at the Research Centre in Heliopolis University for Sustainable Development using a TRACE GC 2000 series gas chromatograph equipped with a flame ionization detector (FID) and SPD-5 fused-silica capillary column (60 m × 0.32 mm, film thickness 1.0 μm). The oven temperature was 60 °C for 1 min, then increased to 250 °C at a rate of 4 °C min^− 1^, then held at 250 °C for 10 min.

The Gas chromatography-mass spectrometry (GC-MS) system (Agilent Technologies) was equipped with a gas chromatograph (7890B) and mass spectrometer detector (5977 A). The analysis was done at the Central Laboratories Network, National Research Centre, Cairo, Egypt. Samples were diluted with hexane (1:19, v/v). The GC was equipped with an HP-5MS column (30 m x 0.25 mm internal diameter and 0.25 μm film thickness) [25].

### Extraction and HPLC Analysis of Apigenin-*7*-*O*-glucoside

Flavonoids including the bioactive secondary metabolite apigenin-*7*-*O*-glucoside (Ap-7-Glc) were extracted from chamomile-dried flowers according to (USP29). The Ap-7-Glc contents in the flowers from different treatments were determined by High-Performance Liquid Chromatography (HPLC) according to the reported method (USP29), which was done also at the Research Centre in Heliopolis University for Sustainable Development. The liquid chromatograph consists of Agilent 1260 Infinity, UniverSil HS C18 PN: UHS18-050905 (250 × 4.6 mm), equipped with a 335-nm detector (Agilent 1100 series).

### Rhizosphere Sampling and Culture-dependent Based Evaluation for Rhizosphere Microbial CFU Counts

The entire root system with the tightly adhering soil of each three plants, from the same treatment (three replicates per treatment), was placed into a flask and resuspended in 15 ml of 0.85% NaCl and vigorously vortexed at the highest speed. The rhizosphere cell suspension was collected, and the vortex treatment was repeated three successive times, each with 15 ml of 0.85% NaCl. A total of 45 ml cell suspension was collected in 50 ml sterilized Falcon tubes [27]. The bacterial and fungal CFU counts, and nitrogen-fixing bacteria (diazotrophs) were assessed on Nutrient agar (NA) (Oxoid Ltd, UK.), Rose Bengal agar (SDFCL, India), and N-deficient combined carbon sources agar (CCM) media, respectively [28]. Serial dilution was performed, and 0.1 ml of the appropriate dilution was spread onto the mentioned media. Each rhizosphere sample was assessed in triplicates. The inoculated plates were incubated at 30℃ for 48–72 h for total bacterial and nitrogen fixing bacteria CFUs. Fungal counts were evaluated after 5 days of incubation and expressed as CFU/g (root dry weight) [29].

### Total Community DNA Extraction and 16 S rRNA Gene PCR Amplification and Illumina MiSeq Sequencing

Pellets were obtained from the remaining rhizosphere microbial cell suspensions, prepared as described above, for each treatment (with *A. circularis* AnHu-treated plants, *Nostoc* sp. NoHu-treated plants, and untreated plants; three replicates per treatment) by centrifugation at 14000 x *g* for 15 min. From each pellet, 0.25 g was subjected to a total community DNA (TC-DNA) extraction (in triplicates) using the DNeasy Power Soil Kit (QIAGEN, Hilden, Germany) according to the manufacturer’s instructions. The concentration and purity of yielded DNA were checked using NanoDrop™ 2000 spectrophotometer and agarose gel electrophoresis. Obtained DNA was diluted 1:10 by milli-Q water and kept at −20°C for further analysis. Bacterial 16S rRNA gene was amplified from the TC-DNA using 16S amplicon PCR forward 5’ TCGTCGGCAGCGTCAGATGTGTATAAGAGACAGCCTACGGGNGGCWGCAG 3’ and reverse 5’ GTCTCGTGGGCTCGGAGATGTGTATAAGAGACAGGACTACHVGGGTATCTAATCC 3’ primers. DNA samples were sequenced using the Illumina MiSeq platform (Illumina, San Diego, CA, USA) to target the V3–V4 region of the 16 S rRNA gene (IGATech. Italy). The data of high-throughput sequencing of the three treatments (three replicates per each) have been submitted to the Sequence Read Archive (SRA) (http://gsa.big.ac.cn/) of the National Center of Biotechnology Information (NCBI) under BioProject accession number PRJNA1186981.

### Data Processing and Statistical Analysis

Raw sequence reads were initially processed using galaxy web platform public server (https://usegalaxy.org) [30] following the default settings, based on the Standard Operating Procedure (SOP) for MiSeq data [31]. Paired-end mating was performed with a minimum overlap length of 50 bp, maximum mismatches threshold of 15, and the sequences with quality score < 30 were excluded. The remaining sequences were clustered into Operational Taxonomic Units (OTUs) at a 97% similarity cutoff. The representative OTU sequences were identified using the GreenGenes classifier (http://greengenes.lbl.gov/) [32]. The OTUs that contained only single sequence across all samples were removed from the subsequent analyses. Extreme replicates were excluded from the analysis due to insufficient sequencing depth (< 5,000 reads) and their pronounced divergence from the remaining biological replicates in beta-diversity and principal coordinate analysis (PCoA) clustering patterns. Alpha-diversity was estimated using Chao1 estimator, ACE index, Shannon diversity index, and Simpson index. Significant differences between diversity indices among the treatments were determined using the Kruskal–Wallis rank-sum test. Beta-diversity was visualized using principal coordinate analysis (PCoA) using the “vegan” package in R v.4.1.0 (https://new.microbiomeanalyst.ca) [33, 34]. Differences in beta-diversity were tested using repeated measures permutational multivariate analysis of variance (PERMANOVA) based on Bray–Curtis dissimilarity [35, 36]. Dendrogram analyses at all feature levels were also generated (https://new.microbiomeanalyst.ca). Venn diagrams were generated using EVenn platform (http://www.ehbio.com/test/venn) [37]. The linear discriminant analysis (LDA) effect size (LEfSe) method was performed using MicrobiomeAnalyst platform with a significance threshold of *p* < 0.05 (FDR-adjusted) and an LDA score cutoff ≥ 2.0. Significant changes in the relative abundance of dominant taxa were determined using ANOVA, followed by Tukey’s honest significance detection test (*p* < 0.05) using R language software (http://www.r.project.org). Statistical analyses of plant growth related parameters and microbial CFU counts were performed using STATISTICA software version 10.0.

## Results

### Impact of Cyanobacterial Application on Chamomile Growth Performance

**Fig. 1:**
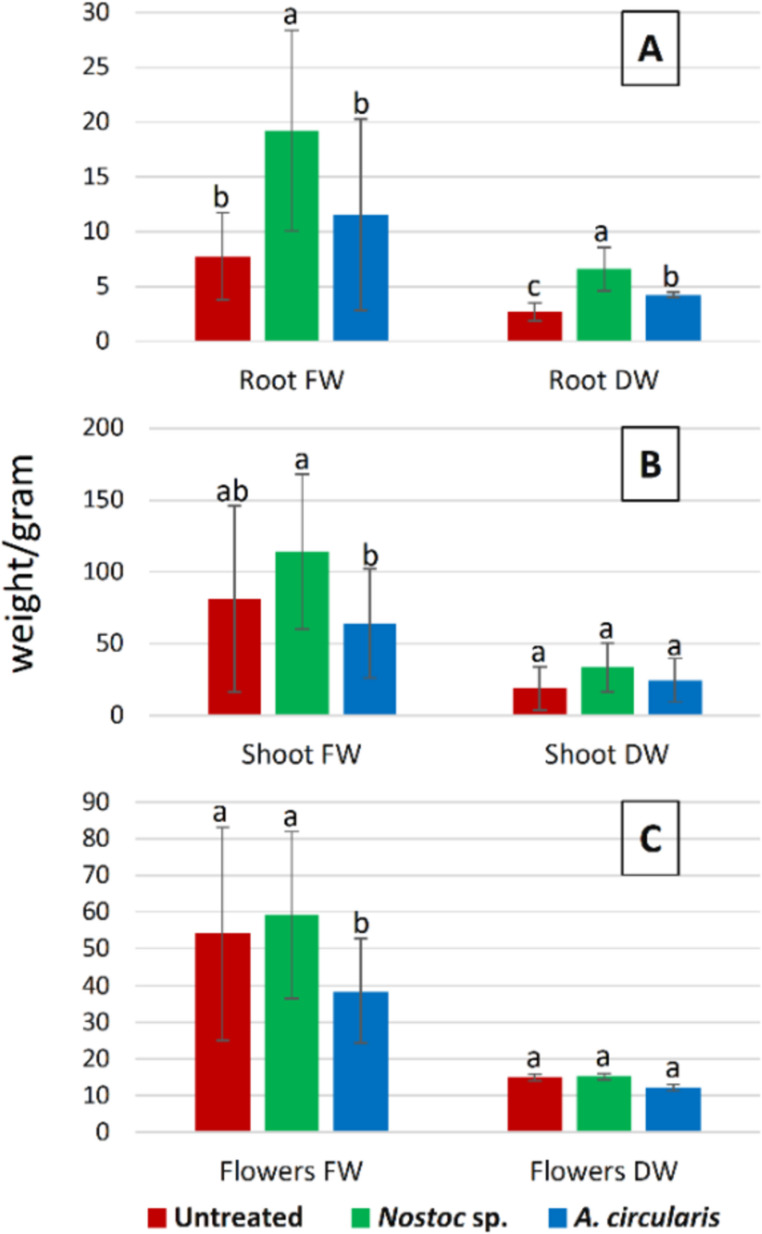
Fresh weight (FW) and dry weight (DW) of the root **(A)**, shoot **(B)** and flowers **(C)** of chamomile plant treated with cyanobacteria *Nostoc* sp. NoHu or *A*. *circularis* AnHu strains compared to untreated plants. Different litters indicate significant differences (*p* value < 0.05)

The impact of *Nostoc* sp. NoHu and *A. circularis* AnHu strains on chamomile growth was assessed via evaluating the fresh and dry weights of chamomile roots, shoots, and flowers compared to untreated plants. A significantly higher root fresh weight was recorded for chamomile plants treated with *Nostoc* sp. (19.21 g) compared to those treated with *A*. *circularis* (11.51 g) and untreated (7.73 g) plants (Fig. 1A). Root dry weight was significantly higher in the plants treated with either *Nostoc* sp. or *A*. *circularis* (6.57 and 4.12 g, respectively) compared to untreated (2.67 g) plants (Fig. 1A). *Nostoc* sp.-treated plants exhibited a significantly higher shoot fresh weight (113.96 g) than untreated (81.19 g) and *A*. *circularis*-treated (64.07 g) plants (Fig. 1B), while no significant differences were observed in shoot dry weight among the treated and untreated plants (Fig. 1B). In addition, the calculated shoot-to-root ratio showed lower values for both *Nostoc* sp.-treated (5.63), and *A*. *circularis*-treated plants (5.91) compared to untreated plants (7.09).

The fresh weight of yielded flowers was also evaluated as affected by the different treatments. While flowers from *Nostoc* sp.-treated plants tended to exhibit a higher fresh weight (59.24 g), this difference was not statistically significant compared to untreated (54.17 g) plants (Fig. 1C). Conversely, flowers from *A*. *circularis*-treated plants showed a significantly lower fresh weight (38.47 g) (Fig. 1C).

### Impact of Cyanobacterial Application on the Active Constituents of Chamomile Essential Oil

The obtained essential oil, through hydro-distillation using a standard Clevenger apparatus, varied across the different treatments. *Nostoc* sp.-treated plants produced a deep blue essential oil, whereas green color was observed for untreated and *A*. *circularis*-treated plants (Fig. S1). Gas chromatography (GC) analysis revealed that the highest essential oil was yielded from the flowers collected from *Nostoc* sp.-treated plants (0.28% v/w), followed by untreated plants (0.22% v/w), with *A*. *circularis*-treated plants yielding nearly half the amount (0.14% v/w).

GS-MS-based chemical analysis for the essential oils obtained from cyanobacteria-treated and untreated plants resulted in identifying almost 27 compounds (Figure S2, and Table S1A, B, and C). The quality characteristics of chamomile essential oil, determined based on specific marker constituents, also varied across the treatments (Table 1). The contents of (E)-β-farnesene, α-bisabolol oxide B, α-bisabolol oxide A, and chamazulene were higher in *Nostoc* sp.-treated plants (1.39, 10.3, 28.85, and 1.24%, respectively) compared to the untreated plants (0.99, 8.25, 22.05, and 0.46%, respectively). Conversely, α-bisabolone oxide A and α-bisabolol were higher in untreated plants (3.99 and 0.56%, respectively) than in the *Nostoc* sp.-treated plants (3.53 and 0.42%, respectively). Flowers from *A*. *circularis*-treated plants showed the lowest levels of all evaluated marker constituents (Table 1).

**Table 1:** GC-MS-based chemical analysis for the content of quality key markers of essential oil content obtained by the hydro-distillation from chamomile plants treated with cyanobacteria strains *Nostoc* sp. NoHu or *A*. *circularis* AnHu compared to untreated plants. R_t_; the retention time

No.	*R*_t_	Compound	Treatments		
			Untreated	Nostoc sp.	A. circularis
1	9.7	(E)-β-farnesene %	0.99	1.39	0.56
2	14.5	Spathulenol %	3.2	3.13	2.21
3	14.7	α-bisabolol oxide B %	8.25	10.3	6.23
4	15.0	α-bisabolone oxide A %	3.99	3.53	3.51
5	15.4	α-bisabolol %	0.56	0.42	0.21
6	17.3	α-bisabolol oxide A %	22.05	28.85	19.14
7	16.7	Chamazulene %	0.46	1.24	0.29

### Cyanobacterial Application Increased Ap-7-Glc Content in Chamomile Flowers

Apigenin-7-O-glucoside (Ap-7-Glc) was extracted from the flowers of cyanobacteria- treated and untreated plants. HPLC-based analysis revealed that both cyanobacterial strains significantly increased Ap-7-Glc content in chamomile flowers compared to untreated plants. Amongst, *Nostoc* sp. treatment displayed the significantly highest Ap-7-Glc content (0.45%) compared to *A*. *circularis* treatment (0.25%) that was closer to the untreated plants (0.22%) (Figure S3).

### Biplot Principal Component Analysis

**Fig. 2:**
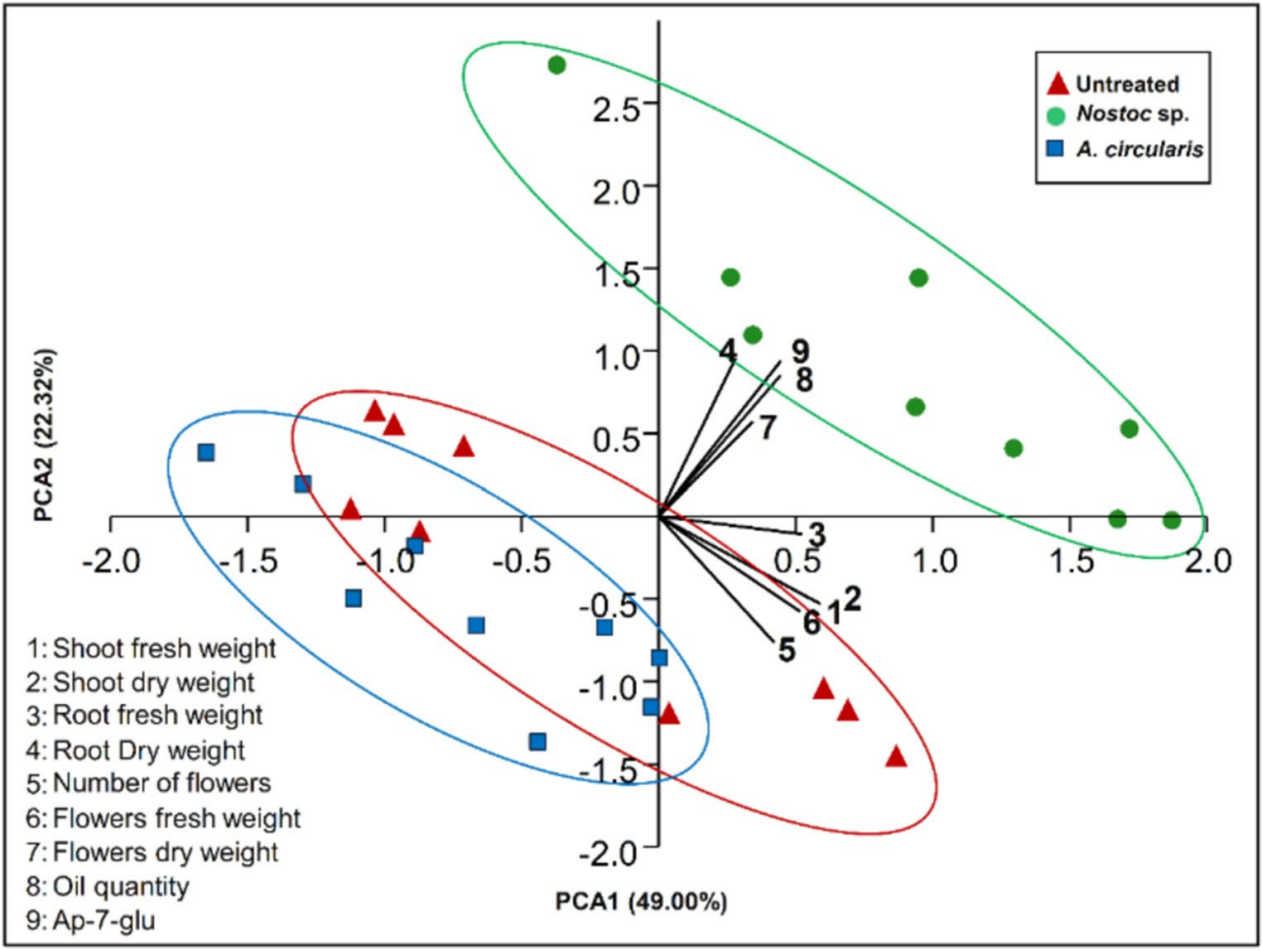
Biplot principal component analysis (PCA) for PC1 and PC2 showing the correlation between the tested plant growth related parameters (fresh and dry weights of roots, shoots and flowers, number of flowers, oil quality, and apigenin) and treatments (*Nostoc* sp., *A*. *circularis* and untreated plants). Arrows with numbers indicate the tested parameters. Colored circles around the shapes represent the treatments samples

To explore the link between the tested parameters, including the plant shoot, root, and flowers fresh and dry weights, essential oil yield, and Ap-7-Glc content, with a certain treatment, a biplot principal component analysis (PCA) was performed. As shown in Fig. 2, the first two principal components (PCA 1 and PCA 2) captured 71.32% of the total variation in the tested parameters as they explained 49% and 22.32% of the variance, respectively. Samples from *Nostoc* sp.-treated plants were distinctly separated away from those of *A. circularis*-treated and untreated plants. In addition, most of the growth-related parameters, such as root dry weight, flower dry weight, oil quantity, and Ap-7-Glc content, were positively associated with the *Nostoc* sp. treatment (Fig. 2). In contrast, *A*. *circularis* treated samples clustered apparat from *Nostoc* sp.-treated samples with some overlap observed with untreated plant samples.

### Impact of Cyanobacterial Applications on Bacterial and Fungal Colony-forming Unit Counts

The colony-forming unit (CFU) counts of the tested microbial populations differed significantly across the treatments. Rhizosphere soil from *A*. *circularis*-treated plants exhibited the highest CFU counts for both bacteria and diazotrophs, followed by the untreated plants, while *Nostoc* sp.-treated plants displayed the lowest counts. Fungal CFU counts followed a similar pattern, with significant differences observed only between the *A*. *circularis* and *Nostoc* sp. treatments (Figure S4), indicating that the rhizosphere of *A*. *circularis*-treated plants harbored the highest population densities.

### Cyanobacterial Inoculation Shifted Chamomile Rhizosphere Bacterial Community Composition

Amplicon sequencing of the bacterial community was performed for the rhizosphere soil collected from both untreated and cyanobacteria-treated chamomile plants. The analysis yielded a total of 916,555 sequences, generated from the nine composite rhizosphere samples after read-quality filtering with an average of 31,509 sequences per sample. Bacterial sequences were clustered into 1130 OTUs at a 97% sequence similarity level. After merging the minor taxa with counts less than ten, the remaining sequences were affiliated into 31 phyla, 72 classes, 107 orders, 169 families, 164 genera, and 30 species.

Beta-diversity using principles coordinate analysis (PCoA) revealed that the bacterial community from *Nostoc* sp.-treated plants was distinctly separated from that of *A*. *circularis*-treated plants along the PCoA axis1, which explained 50.2% of the variation in community composition (Figure S5). The community from *A*. *circularis*-treated plants was separated away from untreated plant community along axis2, which accounted for 10.4% of the variation (Figure S5). These results indicate that the bacterial community from *A*. *circularis*-treated plants differed from both untreated and *Nostoc* sp.-treated plants. The analysis was conducted after excluding the extreme replicates (Figure S5).

**Fig. 3:**
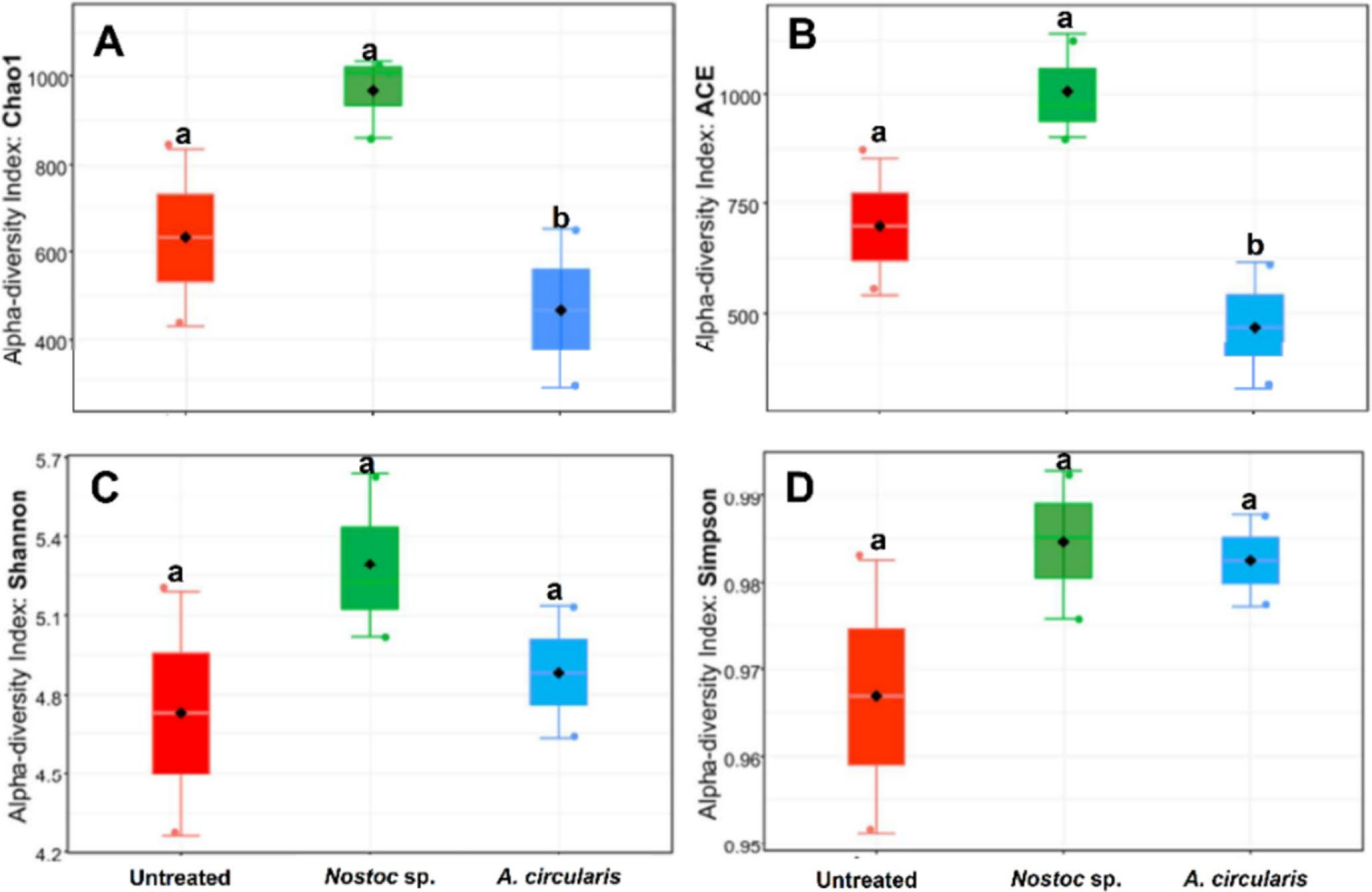
Alpha diversity analysis comparing between the bacterial community in the rhizosphere of cyanobacteria strains *Nostoc* sp. NoHu and *A*. *circularis* AnHu-treated plants and untreated based on the Chao1 **(A)**, ACE **(B)**, Shannon **(C)**, and Simpson **(D)** indices using the 16 S amplicon data. Different letters in the top of bars in panels indicate significant difference among treatments, *p* value < 0.05; One-way ANOVA followed by Tukey’s multiple comparison test. The center value represents the median of alpha index. Points represent random variation on the location of each point

Alpha-diversity indices provided insights into the richness and diversity of the bacteria communities across the tested samples. Based on Chao1 and ACE indices, the *Nostoc* sp.-treated samples exhibited the highest bacterial abundance, followed by the untreated samples, while the *A. circularis*-treatment samples showed the lowest abundance (Fig. 3). Statistical analysis revealed significant differences between the *Nostoc* sp. and *A. circularis* treatments. Although the observed differences in the diversity across the samples were not significant, *Nostoc* sp.-treated samples showed the highest diversity, followed by the untreated samples, while *A. circularis*-treated samples displayed the lowest diversity as indicated by Shannon and Simpson indices (Fig. 3).

**Fig. 4:**
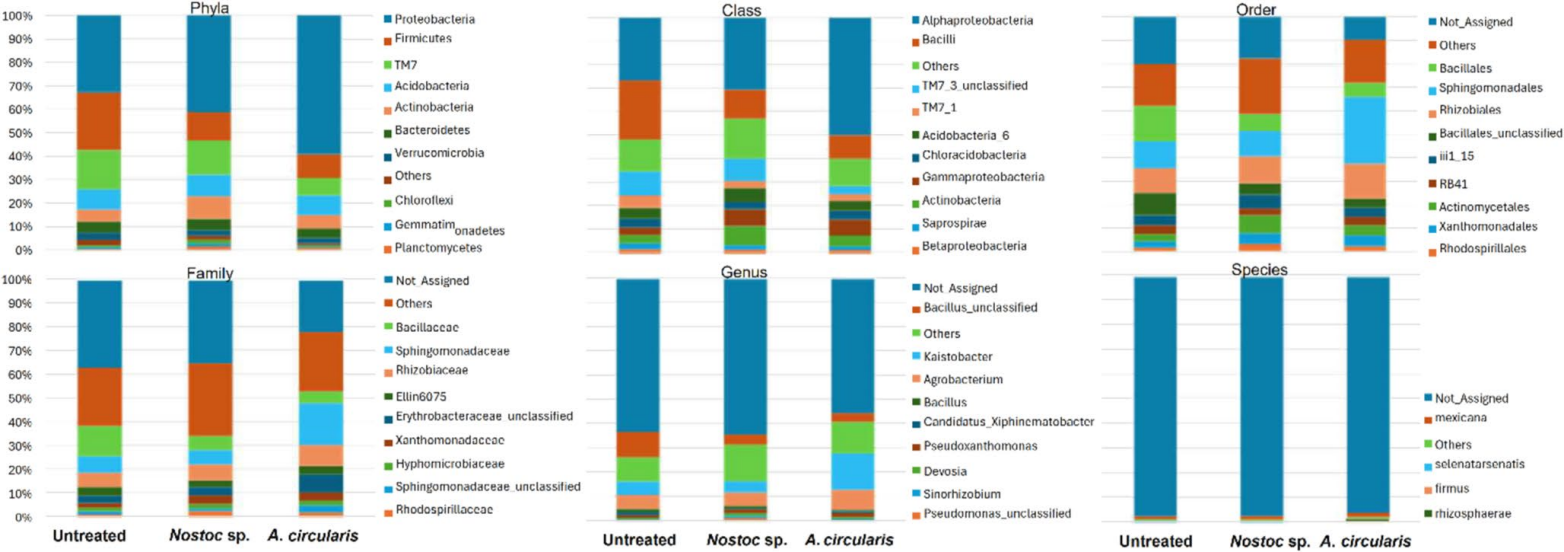
Percentage of relative abundance of most dominant (top ten) bacterial taxa based on 16 S amplicon data from rhizosphere of chamomile plants treated with cyanobacteria strains *Nostoc* sp. NoHu or*A*. *circularis* AnHu compared to untreated plants

Across all samples, bacterial phyla that recorded an average relative abundance greater than 1% included Proteobacteria (44.2%), Firmicutes (15.99%), TM7 (12.83%), Acidobacteria (8.66%), Actinobacteria (6.62%), Bacteroidetes (4.16%), Verrucomicrobia (2.68%), Planctomycetes (1.25%) and Chloroflexi (1.05%), collectively representing 97.8% of the total bacterial abundances (Fig. 4). Proteobacteria, Firmicutes and TM7 phyla showed the highest relative abundances in the rhizosphere samples of both *Nostoc* sp. (41.36, 12.25, 14.37%, respectively) and *A. circularis* (58.79, 10.2, 6.86%, respectively) treatments and untreated samples (32.46, 25.52, 17.28%, respectively) as well (Fig. 4, and Table S2). Among the top ten bacterial phyla (Fig. 4), Proteobacteria showed a significantly higher relative abundance in samples from *A. circularis* treatment compared to untreated samples (Table 2). Phyla such as Bacteroidetes_unclassified, Chlamydiae, Elusimicrobia, Nitrospirae, OD1, and WS3 exhibited relative abundances of less than 1% with significantly lower relative abundances observed in *A. circularis* compared to *Nostoc* sp.-treated samples (Table S2).

**Table 2:**
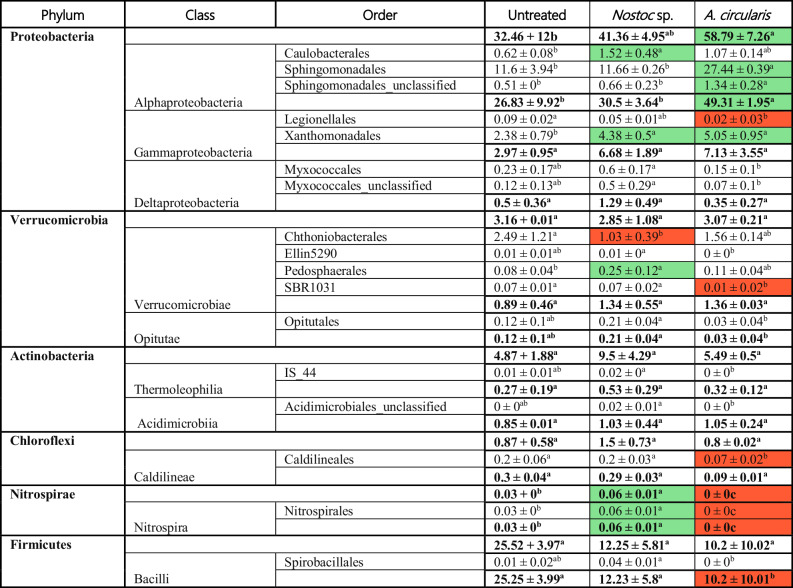
Relative abundance of bacterial orders exhibited significant responses in the rhizosphere of chamomile plant treated with *Nostoc* sp. or *A*. *circularis* cyanobacteria strains and control (untreated plants). In comparison to control plants, the significantly increased orders in the cyanobacteria treated plants are highlighted in green, and the significantly decreased orders are highlighted in red. The relative abundance of the bacterial classes and phyla are shown in bold. Different litters indicate significant differences (*p* value<0.05)

At class level, 14 out of 72 classes showed relative abundances greater than 1%. Among these, Alphaproteobacteria, Acidobacteria_6, Actinobacteria, Bacilli, and TM7_3 showed significant variations across the treatments. Alphaproteobacteria, the most abundant class in all samples, was significantly higher in *A. circularis* treatment (49.31%) relative to untreated (26.83%). In contrast, Bacilli displayed the opposite pattern as it recorded significantly higher abundances in untreated samples (25.25%) than in *A. circularis*-treated (10.2%) samples (Table S3). Among the significantly varied classes with relative abundances less than 1%, except for Gemmatimonadetes_unclassified, all classes exhibited significantly lower abundances in *A. circularis* compared to *Nostoc* sp.-treated and untreated samples (Table S3).

At the order level, 16 orders displayed relative abundances greater than 1%, whereas significant variations were observed in the orders Sphingomonadales (16.9%), Xanthomonadales (3.94%), Caulobacterales (1.07%), and Chthoniobacterales (1.69%) across treatments (Table 2). Sphingomonadales was the most abundant order in the *A. circularis*-treated samples (27.44%), which was significantly higher compared to both untreated (11.6%) and *Nostoc* sp. (11.66%) treated samples (Table 2). On the other hand, Bacillales had the highest relative abundance in untreated samples (15.03%) compared to *Nostoc* sp. (7.9%) and *A. circularis* (5.96%) treated samples (Table 2). Except for Sphingomonadales_unclassified, all orders with abundances less than 1% exhibited significantly lower abundances in *A. circularis*-treated plants compared to *Nostoc* sp.-treated and untreated plants (Table 2).

**Fig. 5:**
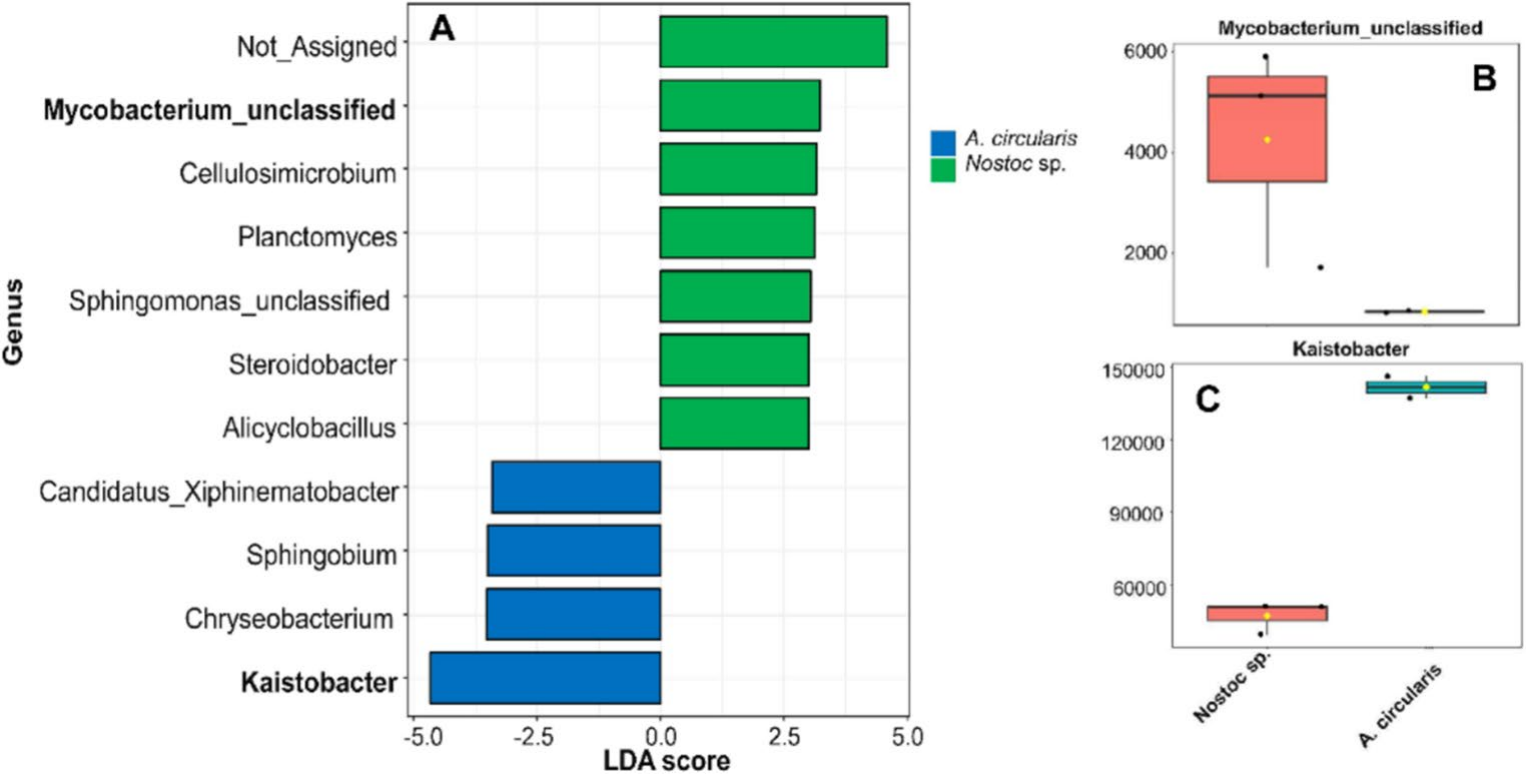
Linear discriminant analysis (LDA) score (**A**) of LEfSe analysis of the rhizosphere bacterial community between chamomile plants treated with *Nostoc* sp. NoHu and *A. circularis* AnHu cyanobacteria strains. Bacterial phylum exhibited the highest LDA score in both treatments are shown in bold. Comparison between the counts of bacterial phyla with the highest LDA score *Mycobacterium_unclassified* **(B)** and *Kaistobacter* **(C)** in both treatments

Among the 14 families with relative abundances greater than 1%, five exhibited significant variations across the treatments. Sphingomonadaceae and Erythrobacteraceae_unclassified were significantly more abundant in *A. circularis*-treated samples (16.49% and 7.71%, respectively) compared to untreated (7.74% and 2.63%, respectively) and *Nostoc* sp.-treated samples (6.54% and 3.46%, respectively). Xanthomonadaceae showed significantly higher abundances in *Nostoc* sp. (3.58%) and *A. circularis* (3.9%) treatments compared to untreated samples (1.82%). Chthoniobacteraceae was more abundant in untreated (2.44%) than *Nostoc* sp. treatment (0.98%), while Sphingomonadaceae_unclassified was significantly more abundant in *A*. *circularis* treatment (3.18%) compared to untreated (1.14%) (Table S4). Among the six most abundant genera, significant differences were observed only for the genus *Kaistobacter*, which showed higher abundances in *A. circularis*-treated samples (14.22%) compared to untreated (6.7%) and *Nostoc* sp.-treated (4.75%) samples (Table 3). Notably, a total of 3 phyla, 3 classes, 9 orders, 24 families, 16 genera, and 5 species were below the detection limit in *A. circularis*-treated samples when compared to the untreated and *Nostoc* sp.-treated samples. Linear Discriminant Analysis (LDA) Effect Size (LEfSe) plot, which highlights microbial taxa that contributed to the differences between the two treatments, *Nostoc* sp. and *A*. circularis, was generated at genus level. It has been shown that 6 and 4 phyla contributed to shaping the community structure of *Nostoc* sp. and *A*. *circularis*, respectively, (Fig. 5A) that could be regarded as biomarkers. However, *Mycobacterium_unclassified* and *Kaistobacter* exhibited the highest LDA score (Fig. 5B and C) *Nostoc* sp. and *A*. *circularis*, respectively. The LDA scores in the other bacterial taxa, including phyla, orders, classes, and families, are shown in figure S6.

**Table 3:**
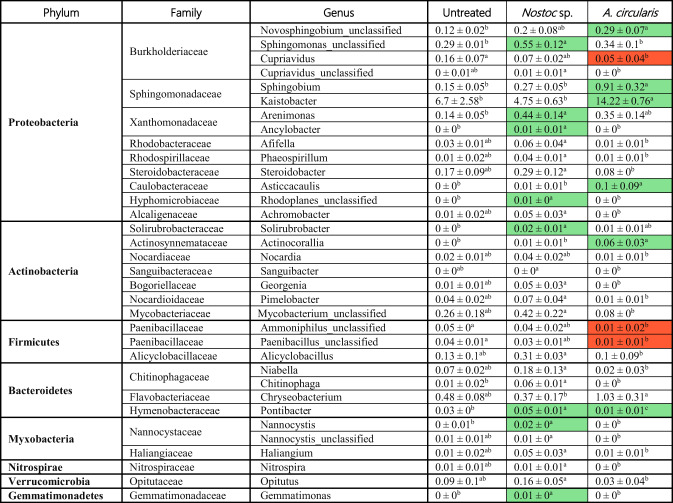
Relative abundance of bacterial genera exhibited significant responses in the rhizosphere of chamomile plant treated with *Nostoc* sp. or *A*. *circularis* cyanobacteria strains and control (untreated plants). In comparison to control plants, the significantly increased orders in the cyanobacteria treated plants are highlighted in green, and the significantly decreased orders are highlighted in red. The phyla and families where the genera are affiliated within are shown. Different litters indicate significant differences (*p* value<0.05)

**Fig. 6:**
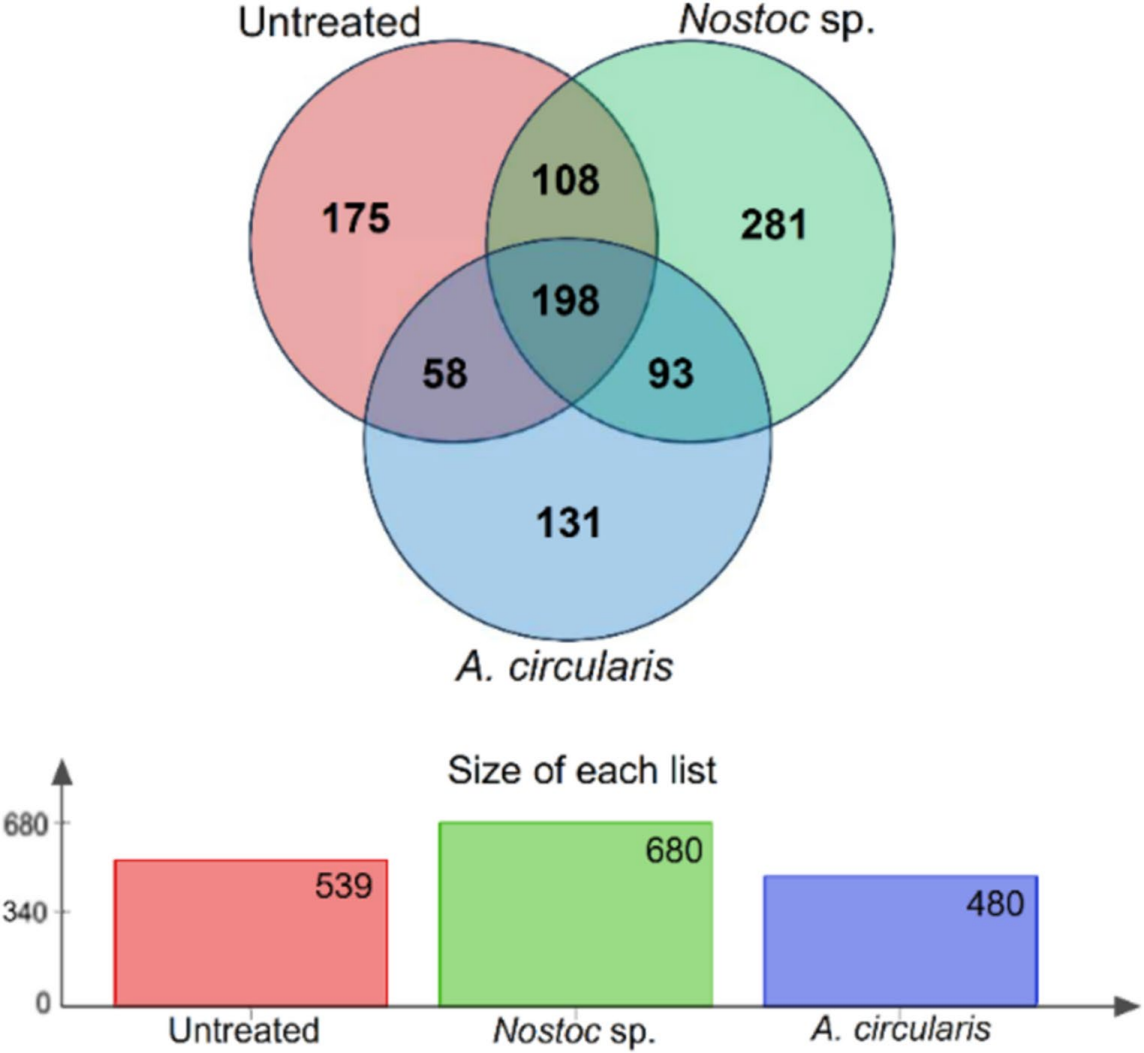
Venn diagram depicting numbers of shared and specific OTUs detected in chamomile rhizosphere treated with cyanobacteria strains *Nostoc* sp. NoHu or*A*. *circularis* AnHu compared to untreated plants

**Fig. 7:**
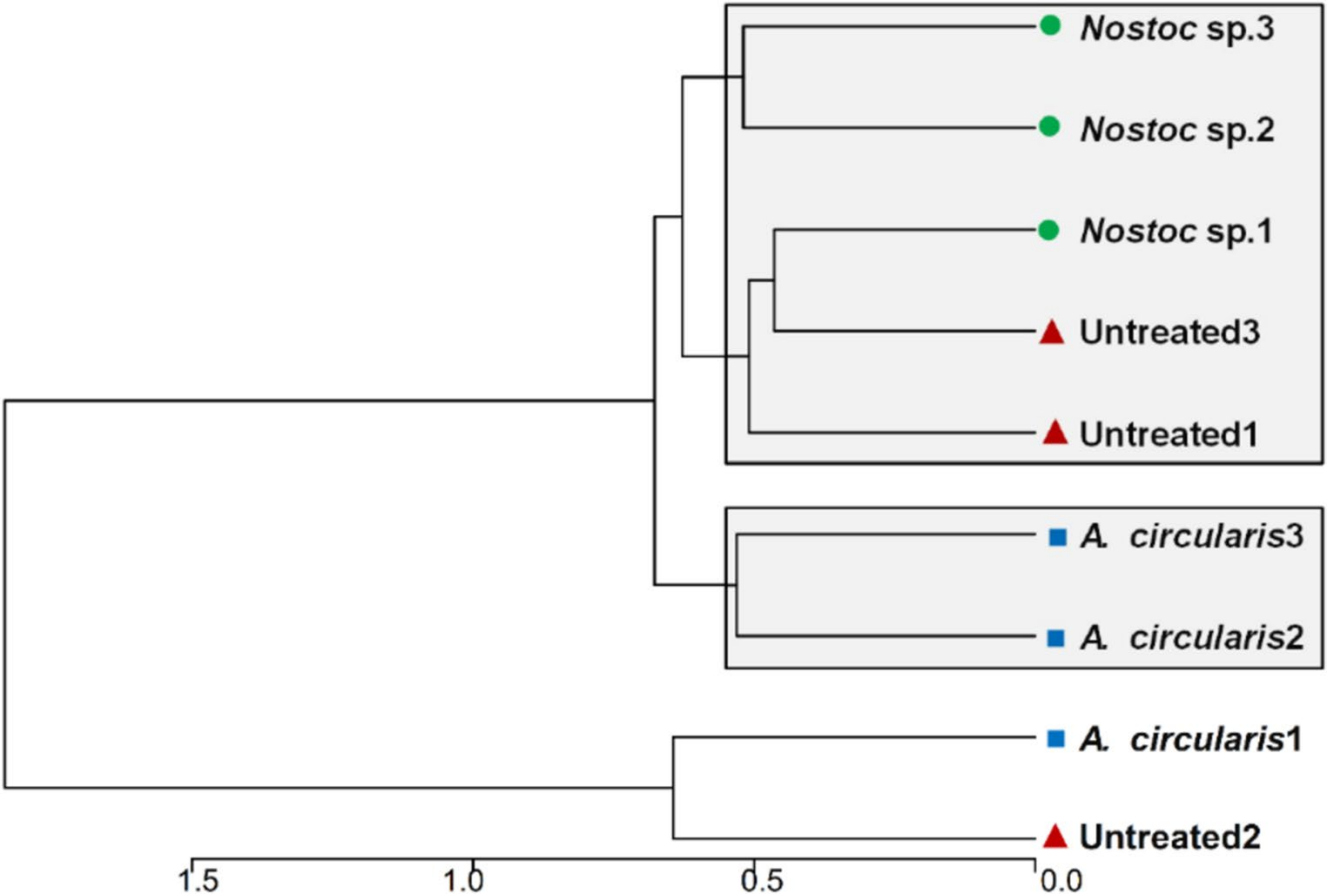
Hierarchical clustering dendrogram of bacterial OTUs based on pairwise Bray-Curtis distance. The dendrogram was generated for 16 S rRNA gene amplicon data from rhizosphere of chamomile plants treated with cyanobacteria strains *Nostoc* sp. NoHu or*A*. *circularis* AnHu compared to untreated plants

Venn diagram, generated at features level, revealed that 198 OTUs were shared between all treatments (Fig. 6). On the other hand, there were 281 and 131 unique OTUs for *Nostoc* sp. and *A. circularis* treatments, respectively, and 175 for untreated samples. However, the OTU number of untreated samples that were shared with *Nostoc* sp. (108 OTUs) is higher than with *A. circularis* (58 OTUs) (Fig. 6). In addition, hierarchical clustering dendrogram at feature level, generated based on pairwise Bray-Curtis distance, with disrespect to the extreme replicas, revealed that untreated and *Nostoc* sp. treated samples were clustered together apart from *A. circularis*-treated samples (Fig. 7).

## Discussion

Although the beneficial effects of cyanobacteria on the growth and productivity of various crops have been widely documented [38], little is known about their mode of action and impact on medicinal plants with regard to secondary bioactive compounds, particularly under field conditions [39]. In the present field study, the application of the *Nostoc* sp. NoHu strain significantly enhanced chamomile plant growth, improved essential oil yield and quality, and increased the apigenin-7-glucoside content, whereas the *A*. *circularis* AnHu strain application exhibited contrasting effects. Moreover, both cyanobacterial strains, *Nostoc* sp. and *A*. *circularis*, displayed distinct shifts in the abundance and diversity of the rhizosphere bacterial community, which were associated with differences in the production of various secondary bioactive compounds in chamomile plants.

In the present study, inoculation with *Nostoc* sp. NoHu inoculation led to a significant improvement in chamomile plant growth performance compared to untreated plants, whereas *A. circularis* AnHu exhibited a negative effect. The contradictory effects observed, despite the ability of both strains to fix atmospheric nitrogen, indicate that nitrogen availability alone does not fully explain the enhanced plant growth, and that additional strain-specific mechanisms are likely contributed to the observed responses. Previous studies have reported that cyanobacteria can produce a wide range of bioactive compounds, including phytohormones such as auxins, gibberellins, cytokinins, as well as vitamins, amino acids, exopolysaccharides, and other plant growth-promoting metabolites [40]. In this context, it is suggested that the beneficial effects associated with *Nostoc* sp., but not *A. circularis*, may be attributed to mechanisms beyond N_2_ fixation. This suggestion is consistent with previous studies showing that even non-nitrogen-fixing cyanobacteria such as *Oscillatoria* sp. and *Phormidium* sp., can also promote the growth of crops such as rice and pea [41, 42]. The growth-promoting effect of these non-nitrogen fixing bacteria has been attributed to the production of bioactive growth-regulating substances, including indole-3-acetic acid (IAA)-like compounds, gibberellin-like substances, cytokinin-like activity, vitamins, and amino acids, which collectively stimulate cell division, enhance nutrient uptake, and improve the plant growth and development, independently of nitrogen fixation [41, 42].

The lower shoot-to-root ratio observed in both *Nostoc* sp. (5.63) and *A. circularis* (5.91) treated plants compared to untreated plants (7.09) indicates a shift in biomass allocation toward root development, which in turn can enhance water and nutrient uptake. Consequently, this improved below-ground development can support overall plant growth and productivity. Comparable responses have been reported in okra (*Abelmoschus esculentus*), where cyanobacterial biofertilizer application increased root biomass and reduced the shoot-to-root ratio, leading to improved nutrient uptake and total plant biomass production [43]. In addition, enhanced root growth and development has been previously reported in chamomile (*Matricaria chamomilla* L.) following the application of *Nostoc carneum*, *Wollea vaginicola*, and *Nostoc punctiforme* cyanobacteria [25].

Chamomile is widely cultivated for the therapeutic and cosmetic properties of its essential oil, making it important to understand how cyanobacterial inoculation impacts both essential oil yield and composition. In the present study, *Nostoc* sp. application increased the essential oil yield (0.28% v/w) compared to untreated plants (0.22% v/w), as well as enhanced the content of vital constituents, including (E)-β-farnesene, α-bisabolol oxide B, α-bisabolol oxide A, and chamazulene. These compounds are recognized as key indicators of the pharmacological and commercial value of chamomile oil, particularly due to their anti-inflammatory properties [44]. The α-bisabolol form is considered as a precursor that can undergo oxidation reactions to form α-bisabolol oxide A and α-bisabolol oxide B. Therefore, the increased levels of α-bisabolol oxidized forms, together with the reduced levels of α-bisabolol, in response to *Nostoc* sp. application may reflect a metabolic shift toward oxidized derivatives rather than precursor compounds. Although the present study did not investigate enzymatic activities, such changes could hypothetically be associated with differential conversion among bisabolol-related compounds, as previously proposed in studies describing the dynamic and flexible nature of the isoprenoid system in aromatic plants [45]. However, these findings suggest that *Nostoc* sp. NoHu application may contribute to enhancing quantitative and qualitative composition of chamomile essential oil through promoting the accumulation of key quality-related compounds. Similarly [25], showed that cyanobacterial strains Nostoc *carneum*, *Wollea vaginicola*, and *Nostoc punctiforme* increased the essential oil content of chamomile (*Matricaria chamomilla* L.) and altered its composition, in a pot experiment. They revealed, through HPLC analysis, that these cyanobacteria were able to produce plant growth–promoting hormones such as indole 3-acetic acid, indole 3-propionic acid and indole 3-butyric acid [25]. Previous molecular studies have also shown that microbial biostimulants can influence the expression of genes associated with terpenoid and phenylpropanoid biosynthesis pathways, thereby affecting secondary metabolites accumulation and composition in medicinal plants [46]. For instance, elevated levels of secondary metabolites have been reported in other medicinal plants such as *Mentha piperita* L. and *Thymus vulgaris* L [16, 47]. In *Mentha piperita* L., cyanobacterial extracts enhanced essential oil accumulation, which was attributed to improved plant growth and metabolic activity [16]. Recently, molecular evidence in *Thymus vulgaris* demonstrated that cyanobacterial inoculation upregulated key genes involved in terpenoid biosynthesis, including 1-deoxy-D-xylulose-5-phosphate reductoisomerase (DXR) and terpene synthase (TPS2), resulting in increased essential oil production [47]. These findings suggest that cyanobacteria enhance secondary metabolite accumulation through a combination of physiological effects (e.g., growth promotion and nutrient uptake) and molecular regulation of biosynthetic pathways responsible for essential oil formation.

In contrast, application of *A. circularis* AnHu led to a reduction in essential oil yield (0.14%) relative to the untreated plants (0.22%), in addition to a decrease in all analyzed key constituents indicating a less compatible interaction of *A. circularis* with chamomile plants under the tested conditions. The contrasting effects on chamomile growth and secondary metabolites production among the two cyanobacteria species further emphasize that the effects of cyanobacteria can be highly species-specific.

Both cyanobacteria species enriched the content of apigenin, a key bioactive component which is considered as another quality marker for chamomile plants. The European Pharmacopoeia stated that chamomile flowers should contain at least 0.25% Ap-7-Glc for therapeutic use, while the U.S. Pharmacopoeia requires that dried chamomile flowers contain not less than 0.3% Ap-7-Glc [48]. Accordingly, *Nostoc* sp. application increased Ap-7-Glc content (0.45%) to exceed the standards set by both the European Pharmacopoeia and the U.S. Pharmacopoeia while *A*. *circularis* (0.25%) meets at least the European Pharmacopoeia standards. These findings strongly support the potential of *Nostoc* sp. as an effective biofertilizer capable of enhancing the medicinal quality of chamomile plants.

In addition to their direct effects on plant physiology, cyanobacteria may indirectly influence plant growth and productivity via altering the rhizosphere microbial community composition is another strategy for influencing plant growth and productivity. Despite that, there is limited knowledge regarding the impact of cyanobacteria on the rhizosphere microbial communities of medicinal plants. In the present study, *Nostoc* sp. application increased both the richness and diversity of chamomile rhizosphere bacterial community compared to untreated plants, as indicated by alpha diversity indexes (Fig. 3), which was accompanied with improved essential oil yield and composition. Accordingly, we proposed that the improved essential oil productivity following *Nostoc* sp. inoculation was not only a direct result of the strain itself but also was mediated by a shift toward beneficial plant-associated bacterial taxa.

*Nostoc* sp. has been widely recognized as a beneficial bacterium capable of establishing symbiotic relationships with a variety of plant species, thereby improving soil health and enhancing plant growth via increasing microbial diversity and activity [49]. In contrast, the reduced richness and diversity in the rhizosphere of the *A*. *circularis*-treated plants was associated with a declines in overall chamomile growth as well as reductions in the content and quality of the chamomile bioactive secondary metabolites. These injurious shifts in the bacterial community may be linked to the potential production of cyanotoxins by *A*. *circularis* through inhibiting the growth and activity of beneficial soil microorganisms [50]. Since cyanotoxin production was not addressed in the present study, further investigations are required to assess its production and to determine its direct effects on the rhizosphere microbiome, to better elucidate the mechanisms underlying the observed responses.

Despite the significant variations in the relative abundances of several bacterial phyla among the treatments, the most abundant phyla remained consistent across all samples, including Proteobacteria (44%), Firmicutes (15.37%), TM7 (12.7%), Acidobacteria (8.97%), Actinobacteria (7.1%), Bacteroidetes (4.3%), Verrucomicrobia (2.68%), Planctomycetes (1.22%), and Chloroflexi (1.16%). Comparable results have been previously reported by [51] in the rhizosphere of chamomile plants. These phyla are also commonly identified as dominant phyla in various soil environments [52, 53], suggesting that cyanobacterial application maintained the overall structural balance of the soil microbial community.

The application of *A*. *circularis* significantly increased the relative abundance of Alphaproteobacteria class, reaching 49.59%, compared to 30.05% and 26.36% in the *Nostoc* sp.-treated and untreated samples, respectively (Table 2). Members of Alphaproteobacteria class have previously been reported to possess pathways and enzymatic processes responsible for microcystin degradation [54], a cyanotoxin known to be produced by *A*. *circularis* [55]. In agreement with this observation, the increased relative abundance of *Sphingobium* and *Novosphingobium*_unclassified genera (Alphaproteobacteria class) may be attributed to their ability to degrade cyanobacteria microcystins, that has been reported by [56]. Although there is no direct evidence demonstrates the involvement of *Kaistobacter* and *Asticcacaulis* genera in microcystins degradation, their affiliation into Alphaproteobacteria class together with their significant enrichment in *A*. *circularis* treated plants suggest a potential degradation role (Fig. 7C). Notably, *Kaistobacter* was the most dominant genus in *A*. *circularis* treatment (14.82%) with a significant prevalence compared to untreated (6.05%) and *Nostoc* sp. treated (4.79%) plants (Table 3). Moreover, *Kaistobacter* also recorded the highest LDA score (Fig. 7A). These results suggest *Kaistobacter* as a biomarker that contributes to shaping the bacterial community in the rhizosphere of *A*. *circularis*-treated plants as well as its potential involvement in microcystin degradation.

Similarly, the significant prevalence of Sphingomonadaceae family and Sphingomonadales order in *A*. *circularis* treatment relative to both untreated and *Nostoc* sp.-treated plants (Table S4 and S5), together with their elevated LDA scores (Figure S6), further indicates their substantial role in shaping the bacterial community in response to *A*. *circularis* application. The enrichment of these specific bacterial taxa was accompanied by a reduction in bacterial diversity, with 6 phyla, 15 classes, 24 orders, 42 families, 36 genera, and 8 species falling below detectable limit in *A*. *circularis*-treated plants compared to untreated and *Nostoc* sp.-treated plants. Collectively, these findings implied that *A*. *circularis* selectively promoted the proliferation of certain bacterial taxa, which may not align with the needs of the chamomile plant, ultimately leading to a negative impact on plant growth and productivity.

Several genera, families, and orders within the Alphaproteobacteria, Betaproteobacteria, Gammaproteobacteria, and Deltaproteobacteria classes were significantly enriched following inoculation with *Nostoc* sp. compared to untreated plants. *Nostoc* sp. are well known for their ability to produce polysaccharides, which are complex carbohydrates that play crucial roles in enhancing soil fertility and promoting plant growth [57]. In this study, *Nostoc* sp. application increased the abundance of various bacterial taxa, many of which are recognized for their capacity to degrade complex organic matter, including polysaccharides. For example, members of the *Arenimonas* and *Chitinophaga* genera are known for their role in breaking down polysaccharides into simpler sugars through the production of extracellular enzymes, making these nutrients more accessible to plants [58]. Similarly, the Opitutaceae family (Verrucomicrobia phylum) is equipped with numerous polysaccharide-degrading pathways and other carbohydrate metabolism strategies [59]. Moreover, *Opitutus* sp. are often found in environments where cyanobacteria are present, such as freshwater and rice rhizospheres [60]. The enrichment of these taxa in response to *Nostoc* sp. application likely plays a vital role in nutrient cycling and availability, creating a nutrient-rich environment that supports chamomile growth and may contribute to the enhanced production of bioactive secondary metabolites. Several studies have demonstrated that rhizosphere microorganisms play an essential role in regulating the accumulation of secondary metabolites in medicinal plants. However, the results suggest that enhanced oil productivity and quality following *Nostoc* sp. inoculation may be mediated by the increased relative abundance and shift toward beneficial plant-associated taxa. Several previous studies have frequently linked the greater rhizosphere microbial diversity with improved nutrient cycling, enhanced microbial cooperation, suppression of plant pathogens, and increased resilience against environmental stress, which collectively may support plant growth and secondary metabolite production [61, 62].

Despite the significant differences in the relative abundance, the bacterial community structure in the rhizosphere of *Nostoc* sp. treatment closely resembles that of untreated samples than to the distinct community observed in *A*. *circularis*-treated plants. This similarity was subsequently reflected in the comparable plant growth performance, essential oil content and quality, and apigenin content observed between *Nostoc* sp.-treated and untreated plants, whereas the results of *A*. *circularis*-treated plants exhibited significantly different responses relative to the untreated plants. Accordingly, these findings imply that the structure and composition of the rhizosphere bacterial community may play an important role in regulating chamomile growth and productivity. However, further studies integrating microbial functional prediction approaches as well as complementary functional assays would provide deeper insights into the ecological roles and metabolic activities underlying these observed plant–microbiome interactions.

## Conclusion

In this study, we showed via a field experiment that, unlike *A*. *circularis* AnHu, *Nostoc* sp. NoHu strain significantly enhanced chamomile plant growth, increased the yield and quality of essential oil, and elevated the apigenin-7-glucoside content. Different cyanobacteria strains exhibited diverged impacts on the rhizosphere bacterial community structure compared to untreated plants, emphasizing the importance of exploring and adopting appropriate cyanobacteria-based biofertilizers. In contrast to *A*. *circularis* AnHu, *Nostoc* sp. increased the bacterial abundance and diversity which in turn improved the content and quality of the bioactive compounds. Thus, *Nostoc* sp. NoHu strain represents a promising candidate bioinoculant for enhancing chamomile productivity. However, these findings are based on a single field site and one growing season, and additional validation across different environments and cultivation cycles would be valuable to confirm their wider applicability.

## Supplementary Information

Below is the link to the electronic supplementary material.

Supplementary Material 1 (DOCX 2.50 MB)

## Data Availability

The datasets generated and/or analyzed during the current study are available from the corresponding author upon reasonable request.
