# Supplementary material for: Shifts in the Rhizosphere Bacterial Community and Improved Essential Oil Yield and Quality in Chamomilla recutita L. Plant Through Cyanobacterial Inoculation
Source: Microb Ecol. 2026 Jun 29;89:133. doi: 10.1007/s00248-026-02815-1 (PMC13315271; doi:10.1007/s00248-026-02815-1)
Supplement: Supplementary file 1 [file 248_2026_2815_MOESM1_ESM.docx]

**Figure S1**. The difference in colour (quality) in the chamomile essential oil extracted by hydro distillation method from the followers of chamomile plants treated with cyanobacteria strains *Nostoc* sp. NoHu or *A*. *circularis* AnHu compared to untreated plants. The bluer the oil, the higher the quality.

**Figure S2.** GC-MS chromatograms for the essential oil obtained by hydro-distillation from chamomile plants treated with cyanobacteria strains *Nostoc* sp. NoHu or *A*. *circularis* AnHu compared to untreated plants.

**Figure S3.** Content (%) of apigenin-7-O-glucoside in the flowers from chamomile plants treated with cyanobacteria strains *Nostoc* sp. NoHu or *A*. *circularis* AnHu compared to untreated plants. Different letters indicate significant differences (*p* value< 0.05).

**Figure S4.** Log numbers of bacteria, diazotrophs and fungi CFU counts in the rhizosphere of chamomile plants treated with cyanobacteria strains *Nostoc* sp. NoHu or *A*. *circularis* AnHu compared to untreated plants. Different letters indicate significant differences (*p* value<0.05).

**Figure S5.** Principal coordinate analysis (PCoA) of weighted UniFrac distances of the rhizosphere bacterial community of chamomile plants treated with cyanobacteria strains *Nostoc* sp. NoHu or *A*. *circularis* AnHu compared to untreated plants.

**Figure S6**. linear discriminant analysis (LDA) score of LEfSe analysis of the rhizosphere bacterial phyla (**A**), order **(B)**, class **(C)**, and family **(D)** comparing between chamomile plants treated with *Nostoc* sp. NoHu and *A. circularis* AnHu cyanobacteria strains. Bacterial taxa exhibited the highest LDA score in both treatments are shown in bold.

**Table S1.** The major constituents (%) of chamomile essential oil at different retention time (R_t_). The oil was obtained from the flowers of cyanobacteria-treated (*Nostoc* sp. NoHu **(A)** or *A*. *circularis* AnHu **(B)**) and untreated plants **(C)** via hydro-distillation.

| ***Nostoc* sp. NoHu (A)** | | | | |
| --- | --- | --- | --- | --- |
| **Peak** | **RT** | **Name** | **Formula** | **Area Sum %** |
| 1 | 2.44 | Undecane | C11H24 | 3.96 |
| 2 | 3.34 | Isopulegol | C10H18O | 1.39 |
| 3 | 3.95 | Dodecane | C12H26 | 5.73 |
| 4 | 5.33 | Tridecane | C13H28 | 0.97 |
| 5 | 9.73 | cis-.beta.-Farnesene | C15H24 | 1.39 |
| 6 | 10.59 | Benzene, (1-butylhexyl)- | C16H26 | 1.07 |
| 7 | 10.74 | Benzene, (1-propylheptyl)- | C16H26 | 0.93 |
| 8 | 11.00 | Benzene, (1-ethyloctyl)- | C16H26 | 0.61 |
| 9 | 11.75 | Benzene, (1-butylheptyl)- | C17H28 | 4.34 |
| 10 | 11.90 | Benzene, (1-propyloctyl)- | C17H28 | 3.98 |
| 11 | 12.18 | Benzene, (1-ethylnonyl)- | C17H28 | 2.51 |
| 12 | 12.86 | Benzene, (1-pentylheptyl)- | C18H30 | 8.37 |
| 13 | 13.31 | Benzene, (1-ethyldecyl)- | C18H30 | 1.56 |
| 14 | 13.94 | Benzene, (1-pentylheptyl)- | C18H30 | 8.64 |
| 15 | 14.48 | Isospathulenol | C15H24O | 3.13 |
| 16 | 14.70 | 2-Furanmethanol, tetrahydro-.alpha.,.alpha.,5-trimethyl-5-(4-methyl-3-cyclohexen-1-yl)-, [2S-[2.alpha.,5.beta.(R*)]]- | C15H26O2 | 10.3 |
| 17 | 14.97 | Bisabolone oxide | C15H24O2 | 3.53 |
| 18 | 15.41 | α-Bisabolol | C15H26O | 0.42 |
| 19 | 16.22 | 2,4-Di-tert-butylphenol | C14H22O | 0.29 |
| 20 | 16.74 | Chamazulene | C14H16 | 1.24 |
| 21 | 17.27 | 2H-Pyran-3-ol, tetrahydro-2,2,6-trimethyl-6-(4-methyl-3-cyclohexen-1-yl)-, [3S-[3.alpha.,6.alpha.(R*)]]- | C15H26O2 | 28.85 |
| 22 | 18.27 | Heptacosane | C27H56 | 0.31 |
| 23 | 20.91 | 1,6-Dioxaspiro[4.4]non-3-ene, 2-(2,4-hexadiynylidene)- | C13H12O2 | 3.88 |
| 24 | 21.09 | n-Hexadecanoic acid | C16H32O2 | 0.64 |
| 25 | 21.95 | (Z)-2-(Hexa-2,4-diyn-1-ylidene)-1,6-dioxaspiro[4.4]non-3-ene | C13H12O2 | 0.33 |
| 26 | 23.01 | Bis(2-ethylhexyl) phthalate | C24H38O4 | 1.62 |

| ***A. circularis* AnHu (B)** | | | | |
| --- | --- | --- | --- | --- |
| **Peak** | **RT** | **Name** | **Formula** | **Area Sum %** |
| 1 | 2.5 | 1-Octanol, 2-butyl- | C12H26O | 7.96 |
| 2 | 3.4 | Isopulegol | C10H18O | 2.85 |
| 3 | 4.0 | Dodecane | C12H26 | 8.42 |
| 4 | 5.4 | Tridecane | C13H28 | 1.57 |
| 5 | 9.7 | cis-.beta.-Farnesene | C15H24 | 0.56 |
| 6 | 10.6 | Benzene, (1-butylhexyl)- | C16H26 | 1.13 |
| 7 | 10.7 | Benzene, (1-propylheptyl)- | C16H26 | 1.3 |
| 8 | 11.0 | Benzene, (1-ethyloctyl)- | C16H26 | 0.82 |
| 9 | 11.7 | Benzene, (1-butylheptyl)- | C17H28 | 6.55 |
| 10 | 11.9 | Benzene, (1-propyloctyl)- | C17H28 | 4.79 |
| 11 | 12.2 | Benzene, (1-ethylnonyl)- | C17H28 | 2.98 |
| 12 | 12.9 | Benzene, (1-pentylheptyl)- | C18H30 | 12.88 |
| 13 | 13.4 | Benzene, (1-ethyldecyl)- | C18H30 | 2 |
| 14 | 13.9 | Benzene, (1-pentylheptyl)- | C18H30 | 5.12 |
| 15 | 14.0 | Benzene, (1-pentylheptyl)- | C18H30 | 3.84 |
| 16 | 14.2 | Benzene, (1-propyldecyl)- | C19H32 | 1.69 |
| 17 | 14.4 | Isospathulenol | C15H24O | 2.21 |
| 18 | 14.7 | 2-Furanmethanol, tetrahydro-.alpha.,.alpha.,5-trimethyl-5-(4-methyl-3-cyclohexen-1-yl)-, [2S-[2.alpha.,5.beta.(R*)]]- | C15H26O2 | 6.23 |
| 19 | 15.0 | Bisabolone oxide | C15H24O2 | 3.51 |
| 20 | 15.4 | α-Bisabolol | C15H26O | 0.21 |
| 21 | 16.2 | 2,4-Di-tert-butylphenol | C14H22O | 0.3 |
| 22 | 16.7 | Chamazulene | C14H16 | 0.29 |
| 23 | 17.3 | 2H-Pyran-3-ol, tetrahydro-2,2,6-trimethyl-6-(4-methyl-3-cyclohexen-1-yl)-, [3S-[3.alpha.,6.alpha.(R*)]]- | C15H26O2 | 19.14 |
| 24 | 18.3 | Heptacosane | C27H56 | 0.28 |
| 25 | 20.9 | 1,6-Dioxaspiro[4.4]non-3-ene, 2-(2,4-hexadiynylidene)- | C13H12O2 | 1.3 |
| 26 | 21.1 | n-Hexadecanoic acid | C16H32O2 | 0.29 |
| 27 | 22.0 | (Z)-2-(Hexa-2,4-diyn-1-ylidene)-1,6-dioxaspiro[4.4]non-3-ene | C13H12O2 | 0.48 |
| 28 | 23.0 | Bis(2-ethylhexyl) phthalate | C24H38O4 | 1.29 |

| **Untreated plants (C)** | | | | |
| --- | --- | --- | --- | --- |
| **Peak** | **RT** | **Name** | **Formula** | **Area Sum %** |
| 1 | 2.466 | 1-Octanol, 2-butyl- | C12H26O | 5.96 |
| 2 | 3.364 | Isopulegol | C10H18O | 2.83 |
| 3 | 4.028 | Dodecane | C12H26 | 7 |
| 4 | 5.351 | Tridecane | C13H28 | 0.82 |
| 5 | 9.734 | cis-.beta.-Farnesene | C15H24 | 0.99 |
| 6 | 10.603 | Benzene, (1-butylhexyl)- | C16H26 | 0.81 |
| 7 | 10.749 | Benzene, (1-propylheptyl)- | C16H26 | 1.03 |
| 8 | 11.023 | Benzene, (1-ethyloctyl)- | C16H26 | 0.57 |
| 9 | 11.78 | Benzene, (1-butylheptyl)- | C17H28 | 5.26 |
| 10 | 11.92 | Benzene, (1-propyloctyl)- | C17H28 | 4.28 |
| 11 | 12.217 | Benzene, (1-ethylnonyl)- | C17H28 | 2.81 |
| 12 | 12.929 | Benzene, (1-pentylheptyl)- | C18H30 | 12.18 |
| 13 | 13.383 | Benzene, (1-ethyldecyl)- | C18H30 | 1.79 |
| 14 | 14.007 | Benzene, (1-pentylheptyl)- | C18H30 | 7.56 |
| 15 | 14.182 | Benzene, (1-propyldecyl)- | C19H32 | 1.32 |
| 16 | 14.508 | Isospathulenol | C15H24O | 3.2 |
| 17 | 14.724 | 2-Furanmethanol, tetrahydro-.alpha.,.alpha.,5-trimethyl-5-(4-methyl-3-cyclohexen-1-yl)-, [2S-[2.alpha.,5.beta.(R*)]]- | C15H26O2 | 8.25 |
| 18 | 15.021 | Bisabolone oxide | C15H24O2 | 3.99 |
| 19 | 15.406 | α-Bisabolol | C15H26O | 0.56 |
| 20 | 16.222 | 2,4-Di-tert-butylphenol | C14H22O | 0.3 |
| 21 | 16.735 | Chamazulene | C14H16 | 0.46 |
| 22 | 17.248 | 2H-Pyran-3-ol, tetrahydro-2,2,6-trimethyl-6-(4-methyl-3-cyclohexen-1-yl)-, [3S-[3.alpha.,6.alpha.(R*)]]- | C15H26O2 | 22.05 |
| 23 | 18.297 | Heptacosane | C27H56 | 0.42 |
| 24 | 20.914 | 1,6-Dioxaspiro[4.4]non-3-ene, 2-(2,4-hexadiynylidene)- | C13H12O2 | 2.58 |
| 25 | 21.1 | n-Hexadecanoic acid | C16H32O2 | 0.63 |
| 26 | 21.975 | (Z)-2-(Hexa-2,4-diyn-1-ylidene)-1,6-dioxaspiro[4.4]non-3-ene | C13H12O2 | 0.67 |
| 27 | 23.024 | Bis(2-ethylhexyl) phthalate | C24H38O4 | 1.7 |

**Table S2.** Relative abundance of bacterial phyla in the rhizosphere of chamomile plant treated with *Nostoc* sp. or *A*. *circularis* cyanobacteria strains compared to untreated plants. In comparison to untreated plants, the significantly increased phyla in the cyanobacteria-treated samples are highlighted in green, and the significantly decreased phyla are highlighted in red. Different letters indicate significant differences (*p* value<0.05).

| **Phylum** | **Untreated** | ***Nostoc* sp.** | ***A. circularis*** |
| --- | --- | --- | --- |
| Acidobacteria | 8.4 ± 2.93^a^ | 9.11 ± 1.84^a^ | 8.49 ± 2.93^a^ |
| TM7 | 17.28 ± 16.25^a^ | 14.37 ± 5.38^a^ | 6.86 ± 3.15^a^ |
| Proteobacteria | 32.46 ± 12^b^ | 41.36 ± 4.95^ab^ | 58.79 ± 7.26^a^ |
| Actinobacteria | 4.87 ± 1.88^a^ | 9.5 ± 4.29^a^ | 5.49 ± 0.5^a^ |
| Bacteroidetes | 4.17 ± 1.23^a^ | 4.27 ± 2.01^a^ | 4.05 ± 2.06^a^ |
| Verrucomicrobia | 3.16 ± 0.01^a^ | 2.85 ± 1.08^a^ | 3.07 ± 0.21^a^ |
| Firmicutes | 25.52 ± 3.97^a^ | 12.25 ± 5.81^a^ | 10.2 ± 10.02^a^ |
| Planctomycetes | 0.93 ± 0.93^a^ | 1.93 ± 0.98^a^ | 0.88 ± 0.56^a^ |
| Chloroflexi | 0.87 ± 0.58^a^ | 1.5 ± 0.73^a^ | 0.8 ± 0.02^a^ |
| Gemmatimonadetes | 0.59 ± 0.57^a^ | 1.44 ± 0.57^a^ | 0.66 ± 0.59^a^ |
| TM7_unclassified | 0.49 ± 0.45^a^ | 0.4 ± 0.07^a^ | 0.16 ± 0.12^a^ |
| Armatimonadetes | 0.22 ± 0.12^a^ | 0.2 ± 0.1^a^ | 0.12 ± 0.06^a^ |
| Not_Assigned | 0.29 ± 0.16^a^ | 0.14 ± 0.05^a^ | 0.1 ± 0.1^a^ |
| Chlorobi | 0.22 ± 0.27^a^ | 0.05 ± 0.02^a^ | 0.09 ± 0.13^a^ |
| Chloroflexi_unclassified | 0.03 ± 0.04^a^ | 0.05 ± 0.03^a^ | 0.07 ± 0.02^a^ |
| Thermi | 0.03 ± 0.04^a^ | 0.07 ± 0.06^a^ | 0.05 ± 0.04^a^ |
| WS2 | 0.03 ± 0.01^a^ | 0.03 ± 0.01^a^ | 0.03 ± 0.05^a^ |
| TM6 | 0.21 ± 0.07^a^ | 0.11 ± 0.09^a^ | 0.02 ± 0.03^a^ |
| Cyanobacteria | 0.06 ± 0.08^a^ | 0.12 ± 0.03^a^ | 0.01 ± 0.02^a^ |
| Parvarchaeota | 0 ± 0^a^ | 0 ± 0.01^a^ | 0.01 ± 0.02^a^ |
| Actinobacteria_unclassified | 0.02 ± 0.03^a^ | 0.03 ± 0.02^a^ | 0.01 ± 0.01^a^ |
| BRC1 | 0.01 ± 0.01^a^ | 0.02 ± 0.01^a^ | 0.01 ± 0.01^a^ |
| NKB19 | 0.01 ± 0.01^a^ | 0.02 ± 0^a^ | 0.01 ± 0.01^a^ |
| Proteobacteria_unclassified | 0.02 ± 0.03^a^ | 0.06 ± 0.06^a^ | 0.01 ± 0.01^a^ |
| Verrucomicrobia_unclassified | 0 ± 0.01^a^ | 0.01 ± 0^a^ | 0.01 ± 0.01^a^ |
| Bacteroidetes_unclassified | 0.01 ± 0.01^ab^ | 0.02 ± 0.01^a^ | 0 ± 0^b^ |
| Chlamydiae | 0 ± 0^b^ | 0.01 ± 0.01^a^ | 0 ± 0^b^ |
| Elusimicrobia | 0 ± 0^b^ | 0.01 ± 0.01^a^ | 0 ± 0^b^ |
| Nitrospirae | 0.03 ± 0^b^ | 0.06 ± 0.01^a^ | 0 ± 0^c^ |
| OD1 | 0.05 ± 0.05^a^ | 0.03 ± 0.03^ab^ | 0 ± 0^b^ |
| WS3 | 0 ± 0^ab^ | 0.01 ± 0.01^a^ | 0 ± 0^b^ |

**Table S3.** Relative abundance of bacterial classes in the rhizosphere of chamomile plant treated with *Nostoc* sp. or *A*. *circularis* cyanobacteria strains compared to untreated plants. In comparison to untreated plants, the significantly increased classes in the cyanobacteria treated samples are highlighted in green, and the significantly decreased classes are highlighted in red. Different letters indicate significant differences (*p* value<0.05).

| **Class** | **Untreated** | ***Nostoc* sp.** | ***A*. *circularis*** |
| --- | --- | --- | --- |
| Gammaproteobacteria | 2.97 ± 0.95^a^ | 6.68 ± 1.89^a^ | 7.13 ± 3.55^a^ |
| Alphaproteobacteria | 26.83 ± 9.92^b^ | 30.5 ± 3.64^b^ | 49.31 ± 1.95^a^ |
| Chloracidobacteria | 4.12 ± 0.56^a^ | 2.93 ± 0.98^a^ | 4.19 ± 1.1^a^ |
| Actinobacteria | 3.67 ± 1.75^b^ | 7.85 ± 3.51^a^ | 4.07 ± 0.21^ab^ |
| Acidobacteria_6 | 3.74 ± 2.06^a^ | 5.51 ± 1.06^a^ | 3.85 ± 1.55^b^ |
| TM7_3_unclassified | 9.69 ± 10.8^a^ | 9.77 ± 5.37^a^ | 3.25 ± 2.06^a^ |
| TM7_1 | 6.07 ± 5.32^a^ | 3.39 ± 1.25^a^ | 2.77 ± 0.78^a^ |
| Bacilli | 25.25 ± 3.99^a^ | 12.23 ± 5.8^a^ | 10.2 ± 10.01^b^ |
| Saprospirae | 1.95 ± 0.94^a^ | 1.81 ± 1^a^ | 1.66 ± 0.97^a^ |
| Spartobacteria | 2.5 ± 1.2^a^ | 1.04 ± 0.39^a^ | 1.56 ± 0.14^a^ |
| Verrucomicrobiae | 0.89 ± 0.46^a^ | 1.34 ± 0.55^a^ | 1.36 ± 0.03^a^ |
| Betaproteobacteria | 1.53 ± 0.46^a^ | 1.91 ± 0.28^a^ | 1.22 ± 0.92^a^ |
| Flavobacteriia | 0.95 ± 0.43^a^ | 0.74 ± 0.47^a^ | 1.17 ± 0.4^a^ |
| Acidimicrobiia | 0.85 ± 0.01^a^ | 1.03 ± 0.44^a^ | 1.05 ± 0.24^a^ |
| Cytophagia | 1.03 ± 0.62^a^ | 1.26 ± 0.35^a^ | 0.98 ± 0.58^a^ |
| TM7_3 | 1.34 ± 0.04^b^ | 1.17 ± 0.03^a^ | 0.8 ± 0.24^c^ |
| Alphaproteobacteria_unclassified | 0.44 ± 0.36^a^ | 0.73 ± 0.27^a^ | 0.54 ± 0.54^a^ |
| Planctomycetia | 0.61 ± 0.64^a^ | 1.14 ± 0.6^a^ | 0.5 ± 0.14^a^ |
| Not_Assigned | 0.96 ± 0.46^a^ | 0.85 ± 0.15^a^ | 0.49 ± 0.22^a^ |
| Phycisphaerae | 0.31 ± 0.26^a^ | 0.77 ± 0.38^a^ | 0.36 ± 0.4^a^ |
| Deltaproteobacteria | 0.5 ± 0.36^a^ | 1.29 ± 0.49^a^ | 0.35 ± 0.27^a^ |
| Thermoleophilia | 0.27 ± 0.19^a^ | 0.53 ± 0.29^a^ | 0.32 ± 0.12^a^ |
| Acidobacteria_6_unclassified | 0.31 ± 0.21^a^ | 0.44 ± 0.18^a^ | 0.28 ± 0.17^a^ |
| Gemm_1 | 0.24 ± 0.23^a^ | 0.5 ± 0.26^a^ | 0.27 ± 0.27^a^ |
| Sphingobacteriia | 0.19 ± 0.08^a^ | 0.4 ± 0.23^a^ | 0.21 ± 0.06^a^ |
| Gitt_GS_136 | 0.12 ± 0.13^a^ | 0.35 ± 0.2^a^ | 0.21 ± 0.05^a^ |
| Ellin6529 | 0.15 ± 0.03^a^ | 0.25 ± 0.17^a^ | 0.19 ± 0.07^a^ |
| Betaproteobacteria_unclassified | 0.1 ± 0.07^a^ | 0.24 ± 0.06^a^ | 0.17 ± 0.12^a^ |
| Chloroflexi | 0.11 ± 0.12^a^ | 0.21 ± 0.15^a^ | 0.15 ± 0.02^a^ |
| Gemmatimonadetes_unclassified | 0.04 ± 0.06^a^ | 0.2 ± 0.13^ab^ | 0.15 ± 0.02^b^ |
| Gemmatimonadetes | 0.18 ± 0.18^a^ | 0.4 ± 0.12^a^ | 0.12 ± 0.17^a^ |
| Solibacteres | 0.2 ± 0.09^a^ | 0.2 ± 0.04^a^ | 0.12 ± 0.17^a^ |
| Fimbriimonadia | 0.22 ± 0.13^a^ | 0.2 ± 0.1^a^ | 0.12 ± 0.06^a^ |
| Pedosphaerae | 0.08 ± 0.04^a^ | 0.25 ± 0.12^a^ | 0.12 ± 0.05^a^ |
| TK17 | 0.05 ± 0.08^a^ | 0.1 ± 0.02^a^ | 0.11 ± 0.08^a^ |
| Anaerolineae | 0.3 ± 0.04^a^ | 0.29 ± 0.03^a^ | 0.09 ± 0.01^a^ |
| SJA_28 | 0.22 ± 0.27^a^ | 0.04 ± 0.01^a^ | 0.06 ± 0.09^a^ |
| Deinococci | 0.03 ± 0.04^a^ | 0.07 ± 0.06^a^ | 0.05 ± 0.04^a^ |
| iii1_8 | 0.01 ± 0.02^a^ | 0.02 ± 0.01^a^ | 0.04 ± 0.06^a^ |
| Gemm_3 | 0.05 ± 0.03^a^ | 0.13 ± 0.05^a^ | 0.03 ± 0.05^a^ |
| MB_A2_108 | 0.03 ± 0^a^ | 0.07 ± 0.05^a^ | 0.03 ± 0.05^a^ |
| S085 | 0.04 ± 0.06^a^ | 0.07 ± 0.01^a^ | 0.03 ± 0.05^a^ |
| SHA_109 | 0.03 ± 0.01^a^ | 0.03 ± 0.01^a^ | 0.03 ± 0.05^a^ |
| Anaerolineae_unclassified | 0.01 ± 0.01^a^ | 0.02 ± 0.02^a^ | 0.03 ± 0.04^a^ |
| OPB56 | 0 ± 0^a^ | 0 ± 0^a^ | 0.03 ± 0.04^a^ |
| Opitutae | 0.12 ± 0.1^ab^ | 0.21 ± 0.04^a^ | 0.03 ± 0.04^b^ |
| Rhodothermi | 0.02 ± 0.03^a^ | 0.04 ± 0.01^a^ | 0.03 ± 0.04^a^ |
| Thermomicrobia | 0.08 ± 0.11^a^ | 0.2 ± 0.14^a^ | 0.03 ± 0.04^a^ |
| Gemm_5 | 0.04 ± 0.02^a^ | 0.11 ± 0.02^a^ | 0.02 ± 0.03^a^ |
| SJA_4 | 0.21 ± 0.07^a^ | 0.11 ± 0.09^ab^ | 0.02 ± 0.03^b^ |
| Parvarchaea | 0 ± 0^a^ | 0 ± 0.01^a^ | 0.01 ± 0.02^a^ |
| Phycisphaerae_unclassified | 0.01 ± 0.01^a^ | 0.02 ± 0.02^a^ | 0.01 ± 0.02^a^ |
| 4C0d_2 | 0.01 ± 0.02^a^ | 0.02 ± 0.02^a^ | 0.01 ± 0.01^a^ |
| Bacilli_unclassified | 0.15 ± 0.22^a^ | 0.02 ± 0.02^a^ | 0.01 ± 0.01^a^ |
| ML635J_21 | 0.02 ± 0.03^a^ | 0.04 ± 0.01^a^ | 0.01 ± 0.01^a^ |
| Others | 0.01 ± 0.01^a^ | 0.01 ± 0.01^a^ | 0.01 ± 0.01^a^ |
| PRR_11 | 0.01 ± 0.01^a^ | 0.02 ± 0.01^a^ | 0.01 ± 0.01^a^ |
| Acidobacteriia | 0 ± 0^a^ | 0 ± 0^a^ | 0 ± 0^a^ |
| Chlamydiia | 0 ± 0^b^ | 0.01 ± 0.01^a^ | 0 ± 0^b^ |
| Elusimicrobia | 0 ± 0^b^ | 0.01 ± 0.01^a^ | 0 ± 0^b^ |
| Gammaproteobacteria_unclassified | 0 ± 0^ab^ | 0.01 ± 0.01^a^ | 0 ± 0^b^ |
| Gemm_2 | 0 ± 0.01^ab^ | 0.02 ± 0.02^a^ | 0 ± 0^b^ |
| Nitriliruptoria | 0 ± 0^b^ | 0.01 ± 0.01^a^ | 0 ± 0^b^ |
| Nitrospira | 0.03 ± 0^b^ | 0.06 ± 0.01^a^ | 0 ± 0^c^ |
| Nostocophycideae | 0.03 ± 0.04^ab^ | 0.05 ± 0.03^a^ | 0 ± 0^b^ |
| Oscillatoriophycideae | 0 ± 0^a^ | 0 ± 0^a^ | 0 ± 0^a^ |
| PRR_12 | 0 ± 0^ab^ | 0.01 ± 0.01^a^ | 0 ± 0^b^ |
| Rubrobacteria | 0 ± 0^ab^ | 0.01 ± 0.01^a^ | 0 ± 0^b^ |
| SC3 | 0.02 ± 0.01^a^ | 0.01 ± 0.01^a^ | 0 ± 0^b^ |
| SM2F11 | 0 ± 0^a^ | 0.01 ± 0.01^a^ | 0 ± 0^a^ |
| Sva0725 | 0 ± 0.01^a^ | 0.01 ± 0.01^a^ | 0 ± 0^a^ |
| TSBW08 | 0 ± 0^ab^ | 0.01 ± 0.01^a^ | 0 ± 0^b^ |

**Table S4.** Relative abundance of bacterial orders in the rhizosphere of chamomile plant treated with *Nostoc* sp. or *A*. *circularis* cyanobacteria strains compared to untreated plants. In comparison to untreated plants, the significantly increased orders in the cyanobacteria-treated samples are highlighted in green, and the significantly decreased orders are highlighted in red. Different letters indicate significant differences (*p* value<0.05).

| **Order** | **Untreated** | ***Nostoc* sp.** | ***A. circularis*** |
| --- | --- | --- | --- |
| Not_Assigned | 19.85 ± 14.38^a^ | 18.23 ± 4.23^a^ | 9.2 ± 1.56^a^ |
| Bacillales | 15.03 ± 1.4^a^ | 7.9 ± 2.79^a^ | 5.96 ± 6.48^a^ |
| Xanthomonadales | 2.38 ± 0.79^b^ | 4.38 ± 0.5^a^ | 5.05 ± 0.95^a^ |
| RB41 | 4.04 ± 0.6^a^ | 2.79 ± 0.97^a^ | 4.07 ± 0.93^a^ |
| Bacillales_unclassified | 9.44 ± 3.65^a^ | 4.24 ± 3.03^a^ | 3.88 ± 4.04^a^ |
| Actinomycetales | 3.37 ± 2.08^a^ | 7.38 ± 3.55^a^ | 3.82 ± 0.13^a^ |
| iii1_15 | 3.63 ± 2.03^a^ | 5.44 ± 1.03^a^ | 3.76 ± 1.42^a^ |
| Sphingomonadales | 11.6 ± 3.94^b^ | 11.66 ± 0.26^b^ | 27.44 ± 0.39^a^ |
| Rhodospirillales | 1.67 ± 0.81^a^ | 3.12 ± 0.89^a^ | 2.38 ± 0.95^a^ |
| Rhizobiales | 11.17 ± 4.32^a^ | 11.17 ± 4.02^a^ | 15.34 ± 0.01^a^ |
| Pseudomonadales | 0.19 ± 0.04^a^ | 1.3 ± 1.35^a^ | 1.73 ± 2.34^a^ |
| Saprospirales | 1.95 ± 0.94^a^ | 1.81 ± 1^a^ | 1.66 ± 0.97^a^ |
| Chthoniobacterales | 2.49 ± 1.21^a^ | 1.03 ± 0.39^b^ | 1.56 ± 0.14^ab^ |
| Verrucomicrobiales | 0.89 ± 0.46^a^ | 1.34 ± 0.55^a^ | 1.36 ± 0.03^a^ |
| Sphingomonadales_unclassified | 0.51 ± 0^b^ | 0.66 ± 0.23^b^ | 1.34 ± 0.28^a^ |
| Flavobacteriales | 0.95 ± 0.43^a^ | 0.73 ± 0.45^a^ | 1.17 ± 0.4^a^ |
| Caulobacterales | 0.62 ± 0.08^b^ | 1.52 ± 0.48^a^ | 1.07 ± 0.14^ab^ |
| Acidimicrobiales | 0.84 ± 0.02^a^ | 1.01 ± 0.44^a^ | 1.05 ± 0.24^a^ |
| Cytophagales | 1.03 ± 0.62^a^ | 1.26 ± 0.35^a^ | 0.98 ± 0.58^a^ |
| Rhizobiales_unclassified | 0.62 ± 0.53^a^ | 1.07 ± 0.21^a^ | 0.78 ± 0.3^a^ |
| Burkholderiales | 1.1 ± 0.19^a^ | 1.17 ± 0.18^a^ | 0.73 ± 0.69^a^ |
| Rhodobacterales | 0.42 ± 0.18^a^ | 0.95 ± 0.42^a^ | 0.6 ± 0.28^a^ |
| Pirellulales | 0.33 ± 0.39^a^ | 0.7 ± 0.41^a^ | 0.42 ± 0.02^a^ |
| Lactobacillales | 0.78 ± 1.07^a^ | 0.09 ± 0.1^a^ | 0.36 ± 0.5^a^ |
| WD2101 | 0.31 ± 0.25^a^ | 0.76 ± 0.39^a^ | 0.36 ± 0.4^a^ |
| I025 | 0.4 ± 0.11^a^ | 0.58 ± 0.36^a^ | 0.27 ± 0.3^a^ |
| Actinomycetales_unclassified | 0.3 ± 0.34^a^ | 0.47 ± 0.24^a^ | 0.24 ± 0.34^a^ |
| Enterobacteriales | 0.1 ± 0.1^a^ | 0.69 ± 0.6^a^ | 0.21 ± 0.07^a^ |
| Sphingobacteriales | 0.19 ± 0.08^a^ | 0.4 ± 0.23^a^ | 0.21 ± 0.06^a^ |
| EW055 | 0.26 ± 0.31^a^ | 0.19 ± 0.16^a^ | 0.16 ± 0.01^a^ |
| Myxococcales | 0.23 ± 0.17^ab^ | 0.6 ± 0.17^a^ | 0.15 ± 0.1^b^ |
| Gaiellales | 0.12 ± 0.02^a^ | 0.14 ± 0.06^a^ | 0.15 ± 0.13^a^ |
| Ellin329 | 0.05 ± 0.04^a^ | 0.1 ± 0.06^a^ | 0.14 ± 0.08^a^ |
| Solibacterales | 0.18 ± 0.06^a^ | 0.17 ± 0.04^a^ | 0.12 ± 0.17^a^ |
| Ellin6067 | 0.08 ± 0^a^ | 0.12 ± 0.01^a^ | 0.12 ± 0.06^a^ |
| Fimbriimonadales | 0.22 ± 0.13^a^ | 0.2 ± 0.1^a^ | 0.12 ± 0.06^a^ |
| MND1 | 0.06 ± 0.05^a^ | 0.13 ± 0.05^a^ | 0.12 ± 0.06^a^ |
| Solirubrobacterales | 0.13 ± 0.18^a^ | 0.28 ± 0.15^a^ | 0.12 ± 0.05^a^ |
| Pedosphaerales | 0.08 ± 0.04^b^ | 0.25 ± 0.12^a^ | 0.11 ± 0.04^ab^ |
| BD7_3 | 0.03 ± 0^a^ | 0.08 ± 0.08^a^ | 0.1 ± 0.09^a^ |
| iii1_15_unclassified | 0.11 ± 0.04^a^ | 0.07 ± 0.03^a^ | 0.09 ± 0.13^a^ |
| Burkholderiales_unclassified | 0.09 ± 0.09^a^ | 0.17 ± 0.04^a^ | 0.07 ± 0.1^a^ |
| Kiloniellales | 0.05 ± 0^a^ | 0.04 ± 0.03^a^ | 0.07 ± 0.1^a^ |
| Myxococcales_unclassified | 0.12 ± 0.13^ab^ | 0.5 ± 0.29^a^ | 0.07 ± 0.1^b^ |
| PK29 | 0.04 ± 0.02^a^ | 0.11 ± 0.04^a^ | 0.07 ± 0.1^a^ |
| Planctomycetales | 0.27 ± 0.23^a^ | 0.35 ± 0.13^a^ | 0.07 ± 0.1^a^ |
| Caldilineales | 0.2 ± 0.06^a^ | 0.2 ± 0.03^a^ | 0.07 ± 0.02^b^ |
| SC_I_84 | 0.06 ± 0.05^a^ | 0.11 ± 0.02^a^ | 0.06 ± 0.09^a^ |
| Thiotrichales | 0.13 ± 0.07^a^ | 0.15 ± 0.01^a^ | 0.06 ± 0.09^a^ |
| Methylophilales | 0.07 ± 0.03^a^ | 0.11 ± 0^a^ | 0.06 ± 0.08^a^ |
| Syntrophobacterales | 0.06 ± 0.02^a^ | 0.06 ± 0.02^a^ | 0.05 ± 0.07^a^ |
| Roseiflexales_unclassified | 0.01 ± 0.02^a^ | 0.02 ± 0.01^a^ | 0.05 ± 0.05^a^ |
| Deinococcales | 0.03 ± 0.04^a^ | 0.07 ± 0.06^a^ | 0.05 ± 0.04^a^ |
| mle1_48 | 0.03 ± 0.04^a^ | 0.06 ± 0.01^a^ | 0.05 ± 0.04^a^ |
| Solirubrobacterales_unclassified | 0.02 ± 0.01^a^ | 0.11 ± 0.09^a^ | 0.05 ± 0.04^a^ |
| AKIW781 | 0.04 ± 0.06^a^ | 0.09 ± 0.04^a^ | 0.04 ± 0.06^a^ |
| Bdellovibrionales | 0.01 ± 0.02^a^ | 0.02 ± 0.01^a^ | 0.04 ± 0.06^a^ |
| Chloroflexales_unclassified | 0 ± 0^a^ | 0.02 ± 0.01^a^ | 0.04 ± 0.06^a^ |
| DS_100 | 0.02 ± 0.01^a^ | 0.02 ± 0.01^a^ | 0.04 ± 0.06^a^ |
| DS_18 | 0.01 ± 0.02^a^ | 0.02 ± 0.01^a^ | 0.04 ± 0.06^a^ |
| Legionellales_unclassified | 0.07 ± 0.05^a^ | 0.05 ± 0.06^a^ | 0.03 ± 0.05^a^ |
| N1423WL | 0.06 ± 0.08^a^ | 0.05 ± 0.02^a^ | 0.03 ± 0.05^a^ |
| Rickettsiales | 0.08 ± 0^a^ | 0.11 ± 0.05^a^ | 0.03 ± 0.05^a^ |
| 0319_7L14 | 0.03 ± 0^a^ | 0.05 ± 0.04^a^ | 0.03 ± 0.04^a^ |
| NB1_j | 0.01 ± 0.01^a^ | 0.04 ± 0.02^a^ | 0.03 ± 0.04^a^ |
| Opitutales | 0.12 ± 0.1^ab^ | 0.21 ± 0.04^a^ | 0.03 ± 0.04^b^ |
| Rhodothermales | 0.02 ± 0.03^a^ | 0.04 ± 0.01^a^ | 0.03 ± 0.04^a^ |
| JG30_KF_CM45 | 0.05 ± 0.07^a^ | 0.16 ± 0.12^a^ | 0.02 ± 0.03^a^ |
| Legionellales | 0.09 ± 0.02^a^ | 0.05 ± 0.01^ab^ | 0.02 ± 0.03^b^ |
| Roseiflexales | 0.04 ± 0.01^a^ | 0.03 ± 0.03^a^ | 0.02 ± 0.03^a^ |
| Alteromonadales | 0.01 ± 0.02^a^ | 0.04 ± 0.03^a^ | 0.01 ± 0.02^a^ |
| Entotheonellales | 0.01 ± 0.01^a^ | 0.02 ± 0.01^a^ | 0.01 ± 0.02^a^ |
| Gemmatales | 0.01 ± 0.01^a^ | 0.09 ± 0.06^a^ | 0.01 ± 0.02^a^ |
| Gemmatimonadales | 0.01 ± 0.01^a^ | 0.02 ± 0.01^a^ | 0.01 ± 0.02^a^ |
| SBR1031 | 0.07 ± 0.01^a^ | 0.07 ± 0.02^a^ | 0.01 ± 0.02^b^ |
| YLA114 | 0 ± 0^a^ | 0 ± 0.01^a^ | 0.01 ± 0.02^a^ |
| 11_24 | 0.02 ± 0.01^a^ | 0.02 ± 0.02^a^ | 0.01 ± 0.01^a^ |
| A31 | 0 ± 0^a^ | 0.01 ± 0^a^ | 0.01 ± 0.01^a^ |
| AKYG1722 | 0.02 ± 0.03^a^ | 0.04 ± 0.02^a^ | 0.01 ± 0.01^a^ |
| Chthoniobacterales_unclassified | 0 ± 0^a^ | 0.01 ± 0^a^ | 0.01 ± 0.01^a^ |
| Others | 0.02 ± 0^a^ | 0.03 ± 0.02^a^ | 0.01 ± 0.01^a^ |
| Pedosphaerales_unclassified | 0 ± 0^a^ | 0.01 ± 0^a^ | 0.01 ± 0.01^a^ |
| SM1D11 | 0.01 ± 0.02^a^ | 0.02 ± 0.02^a^ | 0.01 ± 0.01^a^ |
| Acidimicrobiales_unclassified | 0 ± 0^ab^ | 0.02 ± 0.01^b^ | 0 ± 0^a^ |
| Acidobacteriales | 0 ± 0^a^ | 0 ± 0^a^ | 0 ± 0^a^ |
| Chlamydiales | 0 ± 0^a^ | 0.01 ± 0.01^a^ | 0 ± 0^a^ |
| Chloroflexales | 0.02 ± 0.03^a^ | 0.06 ± 0.06^a^ | 0 ± 0^a^ |
| Chroococcales_unclassified | 0 ± 0^a^ | 0 ± 0^a^ | 0 ± 0^a^ |
| Ellin5290 | 0.01 ± 0.01^ab^ | 0.01 ± 0^a^ | 0 ± 0^b^ |
| envOPS12 | 0 ± 0.01^a^ | 0.01 ± 0.01^a^ | 0 ± 0^a^ |
| Euzebyales | 0 ± 0^a^ | 0.01 ± 0.01^a^ | 0 ± 0^a^ |
| Flavobacteriales_unclassified | 0 ± 0^a^ | 0.02 ± 0.02^a^ | 0 ± 0^a^ |
| Gemmatimonadales_unclassified | 0 ± 0^a^ | 0.01 ± 0.01^a^ | 0 ± 0^a^ |
| IIb | 0 ± 0^a^ | 0.01 ± 0.01^a^ | 0 ± 0^a^ |
| IS_44 | 0.01 ± 0.01^ab^ | 0.02 ± 0^a^ | 0 ± 0^b^ |
| KD8_87 | 0.01 ± 0.02^a^ | 0.02 ± 0.01^a^ | 0 ± 0^a^ |
| MIZ46 | 0.01 ± 0.01^a^ | 0.01 ± 0.01^a^ | 0 ± 0^a^ |
| Nitrospirales | 0.03 ± 0^b^ | 0.06 ± 0.01^a^ | 0 ± 0^c^ |
| Nostocales | 0.03 ± 0.04^a^ | 0.05 ± 0.03^a^ | 0 ± 0^a^ |
| Oceanospirillales | 0 ± 0.01^a^ | 0.01 ± 0.02^a^ | 0 ± 0^a^ |
| Phycisphaerales | 0 ± 0^a^ | 0.01 ± 0.01^a^ | 0 ± 0^a^ |
| Rubrobacterales | 0 ± 0^a^ | 0.01 ± 0.01^a^ | 0 ± 0^a^ |
| S1198 | 0 ± 0^a^ | 0.01 ± 0.01^a^ | 0 ± 0^a^ |
| Sediment_1 | 0 ± 0^a^ | 0.01 ± 0.01^a^ | 0 ± 0^a^ |
| Solibacterales_unclassified | 0.02 ± 0.03^a^ | 0.03 ± 0.01^a^ | 0 ± 0^a^ |
| Spirobacillales | 0.01 ± 0.02^ab^ | 0.04 ± 0.01^a^ | 0 ± 0^b^ |
| Sva0725 | 0 ± 0.01^a^ | 0.01 ± 0.01^a^ | 0 ± 0^a^ |

**Table S5.** Relative abundance of bacterial families in the rhizosphere of chamomile plant treated with *Nostoc* sp. or *A*. *circularis* cyanobacteria strains compared to untreated plants. In comparison to untreated plants, the significantly increased families in the cyanobacteria-treated samples are highlighted in green, and the significantly decreased families are highlighted in red. Different letters indicate significant differences (*p* value<0.05).

| **Family** | **Untreated** | ***Nostoc* sp.** | ***A. circularis*** |
| --- | --- | --- | --- |
| 0319_6A21 | 0.02 ± 0.01^b^ | 0.03 ± 0^a^ | 0 ± 0^c^ |
| Rhizobiaceae | 6.84 ± 1.31^a^ | 6.26 ± 3.75^a^ | 8.74 ± 0.59^a^ |
| Erythrobacteraceae_unclassified | 2.63 ± 0.61^b^ | 3.46 ± 0.43^b^ | 7.71 ± 0.44^a^ |
| Bacillaceae | 12.53 ± 0.23^a^ | 6.3 ± 2.37^a^ | 4.66 ± 5.45^a^ |
| Ellin6075 | 3.96 ± 0.6^a^ | 2.67 ± 0.93^a^ | 3.96 ± 0.78^a^ |
| Xanthomonadaceae | 1.82 ± 0.38^b^ | 3.58 ± 0.53^a^ | 3.9 ± 0.7^a^ |
| Sphingomonadaceae_unclassified | 1.14 ± 0.59^b^ | 1.58 ± 0.02^ab^ | 3.18 ± 1.52^a^ |
| Not_Assigned | 36.52 ± 15.24^a^ | 34.16 ± 2.85^a^ | 21.61 ± 3.73^a^ |
| Rhodospirillaceae | 1.48 ± 0.65^a^ | 2.53 ± 0.53^a^ | 2.22 ± 0.84^a^ |
| Hyphomicrobiaceae | 1.35 ± 0.8^a^ | 1.9 ± 0.61^a^ | 2.18 ± 0.22^a^ |
| Sphingomonadaceae | 7.74 ± 2.69^b^ | 6.54 ± 0.48^b^ | 16.49 ± 0.65^a^ |
| Moraxellaceae | 0 ± 0^a^ | 0.02 ± 0.02^a^ | 1.55 ± 2.2^a^ |
| Chthoniobacteraceae | 2.44 ± 1.21^a^ | 0.98 ± 0.38^b^ | 1.51 ± 0.07^ab^ |
| Bradyrhizobiaceae_unclassified | 0.93 ± 0.4^ab^ | 0.59 ± 0.05^b^ | 1.35 ± 0.54^a^ |
| Rhizobiaceae_unclassified | 0.45 ± 0.33^b^ | 0.41 ± 0.1^b^ | 1.33 ± 0.18^a^ |
| Weeksellaceae | 0.48 ± 0.08^ab^ | 0.37 ± 0.17^b^ | 1.03 ± 0.31^a^ |
| Verrucomicrobiaceae | 0.73 ± 0.28^a^ | 1.1 ± 0.54^a^ | 1.01 ± 0.29^a^ |
| Microbacteriaceae_unclassified | 0.5 ± 0.05^b^ | 0.72 ± 0.18^ab^ | 0.99 ± 0.14^a^ |
| Cytophagaceae | 1 ± 0.57^a^ | 1.22 ± 0.36^a^ | 0.98 ± 0.58^a^ |
| RB40 | 1 ± 0.59^a^ | 1.27 ± 0.38^a^ | 0.94 ± 0.19^a^ |
| Xanthomonadaceae_unclassified | 0.2 ± 0.13^b^ | 0.27 ± 0.03^b^ | 0.92 ± 0.16^a^ |
| Nocardioidaceae | 0.78 ± 0.38^a^ | 1.35 ± 0.48^a^ | 0.92 ± 0.15^a^ |
| Saprospiraceae | 1.18 ± 0.45^a^ | 0.63 ± 0.44^a^ | 0.87 ± 0.66^a^ |
| mb2424 | 0.91 ± 0.41^a^ | 1.09 ± 0.39^a^ | 0.84 ± 0.16^a^ |
| Chitinophagaceae | 0.71 ± 0.44^a^ | 1.1 ± 0.54^a^ | 0.72 ± 0.21^a^ |
| Bacillaceae_unclassified | 1.23 ± 0.53^a^ | 0.6 ± 0.23^a^ | 0.71 ± 0.83^a^ |
| Caulobacteraceae | 0.35 ± 0^b^ | 0.78 ± 0.29^a^ | 0.71 ± 0.02^ab^ |
| Comamonadaceae_unclassified | 0.76 ± 0.09^a^ | 0.79 ± 0.13^a^ | 0.58 ± 0.59^a^ |
| Micromonosporaceae_unclassified | 0.14 ± 0.16^a^ | 0.39 ± 0.24^a^ | 0.46 ± 0.27^a^ |
| Pirellulaceae | 0.29 ± 0.33^a^ | 0.65 ± 0.38^a^ | 0.39 ± 0.02^a^ |
| Hyphomonadaceae | 0.23 ± 0.03^a^ | 0.44 ± 0.2^a^ | 0.38 ± 0.19^a^ |
| Enterococcaceae | 0.78 ± 1.07^a^ | 0.09 ± 0.1^a^ | 0.36 ± 0.5^a^ |
| Caulobacteraceae_unclassified | 0.27 ± 0.07^b^ | 0.73 ± 0.19^a^ | 0.36 ± 0.16^b^ |
| Verrucomicrobiaceae_unclassified | 0.16 ± 0.18^a^ | 0.24 ± 0.03^a^ | 0.35 ± 0.31^a^ |
| Bradyrhizobiaceae | 0.34 ± 0.37^a^ | 0.55 ± 0.25^a^ | 0.28 ± 0.05^a^ |
| Planococcaceae_unclassified | 0.62 ± 0.56^a^ | 0.27 ± 0.15^a^ | 0.27 ± 0.2^a^ |
| Micrococcaceae_unclassified | 0.46 ± 0.5^a^ | 1.15 ± 0.68^a^ | 0.27 ± 0.04^a^ |
| Kineosporiaceae | 0.06 ± 0.01^ab^ | 0.04 ± 0.04^b^ | 0.23 ± 0.14^a^ |
| Sinobacteraceae | 0.35 ± 0.27^a^ | 0.52 ± 0.18^a^ | 0.22 ± 0.08^a^ |
| C111 | 0.25 ± 0.05^a^ | 0.28 ± 0.14^a^ | 0.21 ± 0.07^a^ |
| Phyllobacteriaceae | 0.15 ± 0.09^a^ | 0.1 ± 0.03^a^ | 0.21 ± 0.06^a^ |
| Enterobacteriaceae_unclassified | 0.06 ± 0.05^a^ | 0.21 ± 0.15^a^ | 0.19 ± 0.04^a^ |
| Pseudomonadaceae | 0.18 ± 0.03^a^ | 1.27 ± 1.36^a^ | 0.18 ± 0.14^a^ |
| Sphingobacteriaceae | 0.17 ± 0.08^a^ | 0.31 ± 0.18^a^ | 0.18 ± 0.02^a^ |
| Hyphomicrobiaceae_unclassified | 0.06 ± 0.09^a^ | 0.07 ± 0.05^a^ | 0.17 ± 0.1^a^ |
| Paenibacillaceae | 0.45 ± 0.15^a^ | 0.37 ± 0.09^a^ | 0.16 ± 0.11^b^ |
| Rhodobacteraceae | 0.15 ± 0.14^a^ | 0.38 ± 0.17^a^ | 0.15 ± 0.1^a^ |
| Gaiellaceae | 0.12 ± 0.02^a^ | 0.14 ± 0.06^a^ | 0.15 ± 0.13^a^ |
| Phyllobacteriaceae_unclassified | 0.06 ± 0.04^a^ | 0.1 ± 0.04^a^ | 0.14 ± 0.03^a^ |
| Promicromonosporaceae | 0.19 ± 0.23^a^ | 0.68 ± 0.51^a^ | 0.13 ± 0.18^a^ |
| Microbacteriaceae | 0.09 ± 0.08^a^ | 0.12 ± 0.09^a^ | 0.13 ± 0.16^a^ |
| Fimbriimonadaceae | 0.19 ± 0.12^a^ | 0.16 ± 0.09^a^ | 0.12 ± 0.06^a^ |
| Geodermatophilaceae | 0.04 ± 0.02^a^ | 0.25 ± 0.25^a^ | 0.11 ± 0.08^a^ |
| Alicyclobacillaceae | 0.13 ± 0.1^ab^ | 0.31 ± 0.03^a^ | 0.1 ± 0.09^b^ |
| Flavobacteriaceae | 0.18 ± 0.05^a^ | 0.22 ± 0.21^a^ | 0.1 ± 0.03^a^ |
| Mycobacteriaceae | 0.26 ± 0.18^ab^ | 0.42 ± 0.22^a^ | 0.08 ± 0^b^ |
| Comamonadaceae | 0.11 ± 0.03^a^ | 0.11 ± 0.04^a^ | 0.08 ± 0.11^a^ |
| Iamiaceae_unclassified | 0.01 ± 0.01^b^ | 0.02 ± 0.01^b^ | 0.08 ± 0.01^a^ |
| Acetobacteraceae | 0.06 ± 0.01^a^ | 0.13 ± 0.05^a^ | 0.08 ± 0.01^a^ |
| Nocardioidaceae_unclassified | 0.14 ± 0.2^a^ | 0.34 ± 0.19^a^ | 0.08 ± 0.01^a^ |
| Chitinophagaceae_unclassified | 0.07 ± 0.05^a^ | 0.09 ± 0.03^a^ | 0.07 ± 0.1^a^ |
| Planctomycetaceae | 0.27 ± 0.23^a^ | 0.35 ± 0.13^a^ | 0.07 ± 0.1^a^ |
| Caldilineaceae | 0.18 ± 0.06^a^ | 0.17 ± 0.03^a^ | 0.07 ± 0.02^b^ |
| Thermomonosporaceae | 0.01 ± 0.01^b^ | 0.03 ± 0.01^b^ | 0.07 ± 0.02^a^ |
| Polyangiaceae | 0.04 ± 0.06^a^ | 0.15 ± 0.07^a^ | 0.07 ± 0.02^a^ |
| Rhodobacteraceae_unclassified | 0.04 ± 0.02^a^ | 0.13 ± 0.08^a^ | 0.07 ± 0.02^a^ |
| Piscirickettsiaceae | 0.13 ± 0.07^a^ | 0.15 ± 0.01^a^ | 0.06 ± 0.09^a^ |
| Haliangiaceae | 0.08 ± 0.04^ab^ | 0.21 ± 0.06^a^ | 0.06 ± 0.08^b^ |
| Micromonosporaceae | 0.04 ± 0.06^a^ | 0.17 ± 0.16^a^ | 0.06 ± 0.08^a^ |
| Paenibacillaceae_unclassified | 0.05 ± 0.01^a^ | 0.05 ± 0.02^a^ | 0.06 ± 0.03^a^ |
| Chthoniobacteraceae_unclassified | 0.06 ± 0.01^a^ | 0.05 ± 0.01^a^ | 0.05 ± 0.07^a^ |
| Syntrophobacteraceae | 0.06 ± 0.02^a^ | 0.06 ± 0.02^a^ | 0.05 ± 0.07^a^ |
| Aurantimonadaceae | 0 ± 0^a^ | 0.02 ± 0.01^a^ | 0.05 ± 0.05^a^ |
| Cellulomonadaceae_unclassified | 0.02 ± 0.03^a^ | 0.05 ± 0.03^a^ | 0.05 ± 0.05^a^ |
| EB1017 | 0.01 ± 0.01^a^ | 0.02 ± 0.01^a^ | 0.05 ± 0.05^a^ |
| Streptomycetaceae | 0.1 ± 0.07^a^ | 0.36 ± 0.34^a^ | 0.05 ± 0.05^a^ |
| Erythrobacteraceae | 0.09 ± 0.05^a^ | 0.08 ± 0.02^a^ | 0.05 ± 0.04^a^ |
| Oxalobacteraceae | 0.17 ± 0.09^a^ | 0.1 ± 0.04^a^ | 0.05 ± 0.04^a^ |
| Trueperaceae | 0.03 ± 0.04^a^ | 0.07 ± 0.06^a^ | 0.05 ± 0.04^a^ |
| Ellin517 | 0.07 ± 0.02^ab^ | 0.2 ± 0.11^a^ | 0.04 ± 0.06^b^ |
| AKIW874 | 0 ± 0.01^a^ | 0.03 ± 0.02^a^ | 0.04 ± 0.06^a^ |
| Bdellovibrionaceae | 0.01 ± 0.02^a^ | 0.02 ± 0.01^a^ | 0.04 ± 0.06^a^ |
| Kineosporiaceae_unclassified | 0.2 ± 0.13^a^ | 0.19 ± 0.08^a^ | 0.04 ± 0.06^a^ |
| Methylophilaceae | 0.04 ± 0.02^a^ | 0.04 ± 0.01^a^ | 0.04 ± 0.06^a^ |
| Flavobacteriaceae_unclassified | 0.28 ± 0.32^a^ | 0.12 ± 0.12^a^ | 0.03 ± 0.05^a^ |
| Opitutaceae | 0.1 ± 0.1^ab^ | 0.18 ± 0.05^a^ | 0.03 ± 0.04^b^ |
| Actinosynnemataceae | 0.04 ± 0.05^a^ | 0.08 ± 0.03^a^ | 0.03 ± 0.04^a^ |
| Geodermatophilaceae_unclassified | 0.02 ± 0.03^a^ | 0.22 ± 0.21^a^ | 0.03 ± 0.04^a^ |
| Micrococcaceae | 0.02 ± 0^a^ | 0.03 ± 0.01^a^ | 0.03 ± 0.04^a^ |
| Nocardiaceae | 0.04 ± 0.02^a^ | 0.08 ± 0.01^a^ | 0.03 ± 0.04^a^ |
| Pirellulaceae_unclassified | 0.04 ± 0.05^a^ | 0.05 ± 0.03^a^ | 0.03 ± 0.04^a^ |
| Sphingobacteriaceae_unclassified | 0.02 ± 0^a^ | 0.07 ± 0.06^a^ | 0.03 ± 0.04^a^ |
| Streptomycetaceae_unclassified | 0.08 ± 0.08^a^ | 0.32 ± 0.23^a^ | 0.03 ± 0.04^a^ |
| Oxalobacteraceae_unclassified | 0.04 ± 0.02^b^ | 0.09 ± 0.01^a^ | 0.02 ± 0.03^b^ |
| Acetobacteraceae_unclassified | 0.02 ± 0.03^a^ | 0.1 ± 0.04^a^ | 0.02 ± 0.03^a^ |
| Bryobacteraceae | 0.04 ± 0.02^a^ | 0.01 ± 0.01^a^ | 0.02 ± 0.03^a^ |
| Enterobacteriaceae | 0.04 ± 0.05^a^ | 0.48 ± 0.45^a^ | 0.02 ± 0.03^a^ |
| Iamiaceae | 0.03 ± 0.01^a^ | 0.1 ± 0.07^a^ | 0.02 ± 0.03^a^ |
| Rhodospirillaceae_unclassified | 0.01 ± 0.02^a^ | 0.06 ± 0.04^a^ | 0.02 ± 0.03^a^ |
| Rhodothermaceae | 0.02 ± 0.03^a^ | 0.03 ± 0.01^a^ | 0.02 ± 0.03^a^ |
| A4b | 0.07 ± 0.01^a^ | 0.07 ± 0.02^a^ | 0.01 ± 0.02^b^ |
| Methylophilaceae_unclassified | 0.03 ± 0^b^ | 0.07 ± 0.01^a^ | 0.01 ± 0.02^b^ |
| Brucellaceae | 0.21 ± 0.29^a^ | 0.11 ± 0.04^a^ | 0.01 ± 0.02^a^ |
| Dermabacteraceae | 0 ± 0^a^ | 0.01 ± 0.01^a^ | 0.01 ± 0.02^a^ |
| Ellin5301 | 0 ± 0.01^a^ | 0.01 ± 0.01^a^ | 0.01 ± 0.02^a^ |
| Gemmataceae | 0.01 ± 0.01^a^ | 0.06 ± 0.04^a^ | 0.01 ± 0.02^a^ |
| Methylocystaceae | 0.01 ± 0.02^a^ | 0.04 ± 0.02^a^ | 0.01 ± 0.02^a^ |
| OPB35 | 0 ± 0.01^a^ | 0.01 ± 0^a^ | 0.01 ± 0.02^a^ |
| Pseudonocardiaceae | 0.02 ± 0.03^a^ | 0.07 ± 0.07^a^ | 0.01 ± 0.02^a^ |
| Rhodobiaceae | 0.04 ± 0.01^a^ | 0.07 ± 0.04^a^ | 0.01 ± 0.02^a^ |
| Xanthobacteraceae | 0.01 ± 0.01^a^ | 0.02 ± 0.01^a^ | 0.01 ± 0.02^a^ |
| Nannocystaceae | 0.03 ± 0.04^ab^ | 0.06 ± 0.02^a^ | 0.01 ± 0.01^b^ |
| 211ds20 | 0 ± 0.01^a^ | 0.01 ± 0.01^a^ | 0.01 ± 0.01^a^ |
| Alteromonadaceae | 0.01 ± 0.01^a^ | 0.03 ± 0.02^a^ | 0.01 ± 0.01^a^ |
| Cryomorphaceae | 0.01 ± 0.02^a^ | 0.01 ± 0^a^ | 0.01 ± 0.01^a^ |
| Cystobacteraceae_unclassified | 0 ± 0^a^ | 0.05 ± 0.05^a^ | 0.01 ± 0.01^a^ |
| Entotheonellaceae | 0 ± 0.01^a^ | 0.01 ± 0^a^ | 0.01 ± 0.01^a^ |
| Entotheonellaceae_unclassified | 0 ± 0^a^ | 0.01 ± 0.01^a^ | 0.01 ± 0.01^a^ |
| Gordoniaceae | 0.01 ± 0.01^a^ | 0.02 ± 0^a^ | 0.01 ± 0.01^a^ |
| Intrasporangiaceae_unclassified | 0.01 ± 0.01^a^ | 0.02 ± 0.01^a^ | 0.01 ± 0.01^a^ |
| Legionellaceae | 0.01 ± 0.02^a^ | 0.01 ± 0^a^ | 0.01 ± 0.01^a^ |
| Methylocystaceae_unclassified | 0 ± 0^a^ | 0.01 ± 0.01^a^ | 0.01 ± 0.01^a^ |
| Patulibacteraceae | 0.01 ± 0.01^a^ | 0 ± 0.01^a^ | 0.01 ± 0.01^a^ |
| Rhodothermaceae_unclassified | 0 ± 0^a^ | 0.01 ± 0.01^a^ | 0.01 ± 0.01^a^ |
| S47 | 0 ± 0^a^ | 0.01 ± 0^a^ | 0.01 ± 0.01^a^ |
| Sinobacteraceae_unclassified | 0.01 ± 0.02^a^ | 0.02 ± 0.01^a^ | 0.01 ± 0.01^a^ |
| Solibacteraceae | 0 ± 0^a^ | 0.01 ± 0.01^a^ | 0.01 ± 0.01^a^ |
| Solirubrobacteraceae | 0.01 ± 0.02^a^ | 0.07 ± 0.05^a^ | 0.01 ± 0.01^a^ |
| Caldilineaceae_unclassified | 0.03 ± 0^a^ | 0.02 ± 0^b^ | 0 ± 0^c^ |
| Fimbriimonadaceae_unclassified | 0.03 ± 0^b^ | 0.04 ± 0^a^ | 0 ± 0^c^ |
| Alcaligenaceae | 0.01 ± 0.02^ab^ | 0.05 ± 0.03^a^ | 0 ± 0^b^ |
| Bogoriellaceae | 0.01 ± 0.01^ab^ | 0.05 ± 0.03^a^ | 0 ± 0^b^ |
| Burkholderiaceae | 0.01 ± 0.01^b^ | 0.03 ± 0.01^a^ | 0 ± 0^b^ |
| Cystobacteraceae | 0 ± 0^b^ | 0.01 ± 0^a^ | 0 ± 0^b^ |
| Flammeovirgaceae | 0.02 ± 0.03^ab^ | 0.03 ± 0^a^ | 0 ± 0^b^ |
| Gemmatimonadaceae | 0 ± 0^b^ | 0.01 ± 0^a^ | 0 ± 0^b^ |
| Nitrospiraceae | 0.01 ± 0.01^ab^ | 0.02 ± 0.01^a^ | 0 ± 0^b^ |
| Opitutaceae_unclassified | 0.02 ± 0^a^ | 0.03 ± 0.01^a^ | 0 ± 0^b^ |
| Sanguibacteraceae | 0 ± 0^ab^ | 0 ± 0^a^ | 0 ± 0^b^ |
| Actinosynnemataceae_unclassified | 0 ± 0^a^ | 0.02 ± 0.01^a^ | 0 ± 0^a^ |
| AK1AB1_02E | 0 ± 0^a^ | 0 ± 0^a^ | 0 ± 0^a^ |
| Beutenbergiaceae | 0 ± 0^a^ | 0.01 ± 0^a^ | 0 ± 0^a^ |
| Brucellaceae_unclassified | 0.01 ± 0.01^a^ | 0.03 ± 0.02^a^ | 0 ± 0^a^ |
| Cellulomonadaceae | 0.01 ± 0.02^a^ | 0.07 ± 0.06^a^ | 0 ± 0^a^ |
| Chloroflexaceae | 0.02 ± 0.03^a^ | 0.06 ± 0.06^a^ | 0 ± 0^a^ |
| Conexibacteraceae | 0 ± 0^a^ | 0.01 ± 0.02^a^ | 0 ± 0^a^ |
| Coxiellaceae | 0.01 ± 0.02^a^ | 0.02 ± 0.01^a^ | 0 ± 0^a^ |
| Euzebyaceae | 0 ± 0^a^ | 0.01 ± 0.01^a^ | 0 ± 0^a^ |
| Glycomycetaceae | 0.05 ± 0.07^a^ | 0.05 ± 0.06^a^ | 0 ± 0^a^ |
| Halomonadaceae | 0 ± 0^a^ | 0.01 ± 0.02^a^ | 0 ± 0^a^ |
| Isosphaeraceae | 0 ± 0^a^ | 0.02 ± 0.02^a^ | 0 ± 0^a^ |
| Koribacteraceae_unclassified | 0 ± 0^a^ | 0 ± 0^a^ | 0 ± 0^a^ |
| Kouleothrixaceae | 0.01 ± 0.01^a^ | 0.01 ± 0^a^ | 0 ± 0^a^ |
| Methylobacteriaceae | 0.01 ± 0.02^a^ | 0.02 ± 0.01^a^ | 0 ± 0^a^ |
| mitochondria | 0.01 ± 0.02^a^ | 0.02 ± 0.01^a^ | 0 ± 0^a^ |
| Myxococcaceae | 0 ± 0^a^ | 0.02 ± 0.02^a^ | 0 ± 0^a^ |
| Nocardiaceae_unclassified | 0 ± 0^a^ | 0 ± 0.01^a^ | 0 ± 0^a^ |
| Nostocaceae | 0.02 ± 0.03^a^ | 0.04 ± 0.02^a^ | 0 ± 0^a^ |
| Nostocaceae_unclassified | 0.01 ± 0.01^a^ | 0.01 ± 0.01^a^ | 0 ± 0^a^ |
| Others | 0.02 ± 0.01^a^ | 0.02 ± 0.01^a^ | 0 ± 0^a^ |
| Parachlamydiaceae | 0 ± 0^a^ | 0.01 ± 0.01^a^ | 0 ± 0^a^ |
| PAUC26f | 0.01 ± 0.02^a^ | 0.02 ± 0.01^a^ | 0 ± 0^a^ |
| Polyangiaceae_unclassified | 0 ± 0^a^ | 0.01 ± 0.01^a^ | 0 ± 0^a^ |
| Rhodobiaceae_unclassified | 0 ± 0^a^ | 0.02 ± 0.02^a^ | 0 ± 0^a^ |
| Rickettsiaceae | 0.02 ± 0.02^a^ | 0.02 ± 0.01^a^ | 0 ± 0^a^ |
| Rubrobacteraceae | 0 ± 0^a^ | 0.01 ± 0.01^a^ | 0 ± 0^a^ |
| Sporichthyaceae | 0.01 ± 0.01^a^ | 0.03 ± 0.02^a^ | 0 ± 0^a^ |
| Streptosporangiaceae_unclassified | 0 ± 0^a^ | 0.01 ± 0.01^a^ | 0 ± 0^a^ |
| Thermoactinomycetaceae | 0.01 ± 0.02^a^ | 0.01 ± 0.01^a^ | 0 ± 0^a^ |

**Table S6.** Relative abundance of bacterial genera in the rhizosphere of chamomile plant treated with *Nostoc* sp. or *A*. *circularis* cyanobacteria strains compared to untreated plants. In comparison to untreated plants, the significantly increased genera in the cyanobacteria-treated samples are highlighted in green, and the significantly decreased genera are highlighted in red. Different letters indicate significant differences (*p* value<0.05).

| **Genus** | **Untreated** | ***Nostoc* sp.** | ***A. circularis*** |
| --- | --- | --- | --- |
| *A17* | 0.01 ± 0.02^a^ | 0 ± 0^a^ | 0.01 ± 0.02^a^ |
| *Agrobacterium* | 6.44 ± 1.04^a^ | 5.46 ± 3.4^a^ | 8.01 ± 1.41^a^ |
| *Not_Assigned* | 61.51 ± 8.19^a^ | 63.72 ± 1.87^a^ | 55.92 ± 1.2^a^ |
| *Bacillus_unclassified* | 9.95 ± 0.05^a^ | 4.7 ± 1.87^a^ | 3.44 ± 4.09^a^ |
| *Kaistobacter* | 6.7 ± 2.58^b^ | 4.75 ± 0.63^b^ | 14.22 ± 0.76^a^ |
| *Pseudoxanthomonas* | 1.02 ± 0.01^a^ | 1.43 ± 0^a^ | 1.68 ± 0.77^a^ |
| *Acinetobacter* | 0 ± 0^a^ | 0 ± 0^a^ | 1.36 ± 1.92^a^ |
| *Devosia* | 0.69 ± 0.29^a^ | 1.01 ± 0.22^a^ | 1.05 ± 0.23^a^ |
| *Chryseobacterium* | 0.48 ± 0.08^ab^ | 0.37 ± 0.17^b^ | 1.03 ± 0.31^a^ |
| *Candidatus_Xiphinematobacter* | 1.93 ± 1.54^a^ | 0.46 ± 0.29^a^ | 0.97 ± 0.23^a^ |
| *Sphingobium* | 0.15 ± 0.05^b^ | 0.27 ± 0.05^b^ | 0.91 ± 0.32^a^ |
| *Rhodoplanes* | 0.47 ± 0.4^a^ | 0.61 ± 0.26^a^ | 0.89 ± 0.12^a^ |
| *Luteimonas_unclassified* | 0.14 ± 0.19^a^ | 0.22 ± 0.18^a^ | 0.76 ± 0.65^a^ |
| *Sinorhizobium* | 0.37 ± 0.22^a^ | 0.77 ± 0.36^a^ | 0.73 ± 0.81^a^ |
| *Luteolibacter* | 0.37 ± 0.29^a^ | 0.76 ± 0.49^a^ | 0.62 ± 0.07^a^ |
| *Bacillus* | 1.54 ± 0.35^a^ | 1.03 ± 0.42^a^ | 0.52 ± 0.64^a^ |
| *Aeromicrobium* | 0.43 ± 0.08^a^ | 0.38 ± 0.03^a^ | 0.5 ± 0.14^a^ |
| *Emticicia* | 0.08 ± 0.04^a^ | 0.11 ± 0.12^a^ | 0.41 ± 0.35^a^ |
| *Sphingopyxis* | 0.24 ± 0.11^a^ | 0.36 ± 0.01^a^ | 0.39 ± 0.2^a^ |
| *Enterococcus_unclassified* | 0.78 ± 1.07^a^ | 0.09 ± 0.1^a^ | 0.36 ± 0.5^a^ |
| *Arenimonas* | 0.14 ± 0.05^b^ | 0.44 ± 0.14^a^ | 0.35 ± 0.14^ab^ |
| *Sphingomonas_unclassified* | 0.29 ± 0.01^b^ | 0.55 ± 0.12^a^ | 0.34 ± 0.1^b^ |
| *Novosphingobium_unclassified* | 0.12 ± 0.02^b^ | 0.2 ± 0.08^ab^ | 0.29 ± 0.07^a^ |
| *Balneimonas* | 0.34 ± 0.37^a^ | 0.55 ± 0.25^a^ | 0.28 ± 0.05^a^ |
| *Phenylobacterium* | 0.18 ± 0.03^a^ | 0.45 ± 0.19^a^ | 0.26 ± 0.14^a^ |
| *Psychrobacter_unclassified* | 0 ± 0^a^ | 0.02 ± 0.02^a^ | 0.2 ± 0.28^a^ |
| *Luteimonas* | 0.13 ± 0.11^a^ | 0.6 ± 0.48^a^ | 0.2 ± 0.06^a^ |
| *Pirellula* | 0.08 ± 0.04^a^ | 0.15 ± 0.07^a^ | 0.2 ± 0.06^a^ |
| *Mesorhizobium_unclassified* | 0.11 ± 0.08^a^ | 0.08 ± 0.03^a^ | 0.19 ± 0.03^a^ |
| *Dyadobacter* | 0.17 ± 0.12^a^ | 0.2 ± 0.05^a^ | 0.17 ± 0^a^ |
| *Pseudomonas_unclassified* | 0.15 ± 0.01^a^ | 1.15 ± 1.21^a^ | 0.17 ± 0.13^a^ |
| *Nocardioides* | 0.1 ± 0.07^a^ | 0.23 ± 0.2^a^ | 0.14 ± 0.14^a^ |
| *Mycoplana* | 0.08 ± 0.03^a^ | 0.13 ± 0.04^a^ | 0.14 ± 0.03^a^ |
| *Fimbriimonas* | 0.19 ± 0.12^a^ | 0.16 ± 0.09^a^ | 0.12 ± 0.06^a^ |
| *Promicromonospora* | 0.13 ± 0.14^a^ | 0.36 ± 0.23^a^ | 0.11 ± 0.16^a^ |
| *Alicyclobacillus* | 0.13 ± 0.1^ab^ | 0.31 ± 0.03^a^ | 0.1 ± 0.09^b^ |
| *Asticcacaulis* | 0 ± 0^b^ | 0.01 ± 0.01^b^ | 0.1 ± 0.09^a^ |
| *Flavisolibacter* | 0.14 ± 0.13^a^ | 0.18 ± 0.07^a^ | 0.1 ± 0.09^a^ |
| *Skermanella* | 0.04 ± 0.02^a^ | 0.11 ± 0.05^a^ | 0.1 ± 0.03^a^ |
| *Agromyces* | 0.05 ± 0.04^a^ | 0.08 ± 0.07^a^ | 0.09 ± 0.1^a^ |
| *Mycobacterium_unclassified* | 0.26 ± 0.18^ab^ | 0.42 ± 0.22^a^ | 0.08 ± 0^b^ |
| *Steroidobacter* | 0.17 ± 0.09^ab^ | 0.29 ± 0.12^a^ | 0.08 ± 0^b^ |
| *Flavobacterium* | 0.1 ± 0.04^a^ | 0.16 ± 0.17^a^ | 0.08 ± 0^a^ |
| *Hyphomicrobium* | 0.04 ± 0.06^a^ | 0.09 ± 0.06^a^ | 0.08 ± 0^a^ |
| *Hyphomicrobium_unclassified* | 0.09 ± 0.01^a^ | 0.11 ± 0.06^a^ | 0.08 ± 0.12^a^ |
| *Stenotrophomonas_unclassified* | 0.06 ± 0.05^a^ | 0.26 ± 0.12^a^ | 0.08 ± 0.12^a^ |
| *Pedomicrobium* | 0.05 ± 0.03^a^ | 0.05 ± 0.02^a^ | 0.08 ± 0.01^a^ |
| *Sphingomonas* | 0.04 ± 0.02^a^ | 0.06 ± 0.06^a^ | 0.08 ± 0.01^a^ |
| *Planctomyces* | 0.27 ± 0.23^a^ | 0.35 ± 0.13^a^ | 0.07 ± 0.1^a^ |
| *Lysobacter* | 0.04 ± 0.02^a^ | 0.11 ± 0.04^a^ | 0.07 ± 0.02^a^ |
| *Paracoccus_unclassified* | 0.03 ± 0.05^a^ | 0.11 ± 0.07^a^ | 0.06 ± 0.09^a^ |
| *Ammoniphilus* | 0.22 ± 0.11^a^ | 0.19 ± 0.03^a^ | 0.06 ± 0.08^a^ |
| *Actinocorallia* | 0 ± 0^b^ | 0.01 ± 0.01^b^ | 0.06 ± 0.03^a^ |
| *Geodermatophilus_unclassified* | 0.02 ± 0.03^a^ | 0.2 ± 0.21^a^ | 0.06 ± 0.03^a^ |
| *Niastella* | 0.02 ± 0.03^a^ | 0.03 ± 0^a^ | 0.06 ± 0.03^a^ |
| *Chondromyces* | 0.01 ± 0.01^a^ | 0.02 ± 0.01^a^ | 0.05 ± 0.05^a^ |
| *Geodermatophilus* | 0.02 ± 0.01^a^ | 0.04 ± 0.04^a^ | 0.05 ± 0.05^a^ |
| *Lutibacterium* | 0.01 ± 0.01^a^ | 0.01 ± 0^a^ | 0.05 ± 0.05^a^ |
| *Paracoccus* | 0.05 ± 0.03^a^ | 0.11 ± 0.06^a^ | 0.05 ± 0.05^a^ |
| *Streptomyces_unclassified* | 0.1 ± 0.07^a^ | 0.36 ± 0.34^a^ | 0.05 ± 0.05^a^ |
| *Virgibacillus_unclassified* | 0.13 ± 0.12^a^ | 0.03 ± 0.01^a^ | 0.05 ± 0.05^a^ |
| *Cupriavidus* | 0.16 ± 0.07^a^ | 0.07 ± 0.02^ab^ | 0.05 ± 0.04^b^ |
| *Sphingobacterium* | 0.03 ± 0.01^a^ | 0.1 ± 0.1^a^ | 0.05 ± 0.04^a^ |
| *Terribacillus* | 0.14 ± 0.02^a^ | 0.25 ± 0.18^a^ | 0.05 ± 0.04^a^ |
| *Agromyces_unclassified* | 0.03 ± 0.05^a^ | 0.04 ± 0.02^a^ | 0.04 ± 0.06^a^ |
| *Bdellovibrio* | 0.01 ± 0.02^a^ | 0.02 ± 0.01^a^ | 0.04 ± 0.06^a^ |
| *Brevibacillus* | 0.03 ± 0^a^ | 0.02 ± 0.01^a^ | 0.04 ± 0.06^a^ |
| *Caulobacter* | 0.01 ± 0.02^a^ | 0.01 ± 0^a^ | 0.04 ± 0.06^a^ |
| *Pedobacter* | 0.02 ± 0.01^a^ | 0.06 ± 0.06^a^ | 0.04 ± 0.06^a^ |
| *Rubrivivax* | 0.04 ± 0.02^a^ | 0.06 ± 0.01^a^ | 0.04 ± 0.06^a^ |
| *Truepera* | 0.01 ± 0.02^a^ | 0.02 ± 0.01^a^ | 0.04 ± 0.06^a^ |
| *Actinoplanes* | 0.03 ± 0.04^a^ | 0.12 ± 0.12^a^ | 0.03 ± 0.05^a^ |
| *Paenibacillus* | 0.08 ± 0.04^a^ | 0.05 ± 0.01^a^ | 0.03 ± 0.05^a^ |
| *Opitutus* | 0.09 ± 0.1^ab^ | 0.16 ± 0.05^a^ | 0.03 ± 0.04^b^ |
| *Lentzea_unclassified* | 0.04 ± 0.05^a^ | 0.08 ± 0.03^a^ | 0.03 ± 0.04^a^ |
| *Methylobacillus* | 0.01 ± 0.02^a^ | 0.03 ± 0.01^a^ | 0.03 ± 0.04^a^ |
| *Niabella* | 0.07 ± 0.02^ab^ | 0.18 ± 0.13^a^ | 0.02 ± 0.03^b^ |
| *Amaricoccus* | 0.02 ± 0.02^a^ | 0.06 ± 0.04^a^ | 0.02 ± 0.03^a^ |
| *Aminobacter* | 0.03 ± 0.01^a^ | 0.02 ± 0^a^ | 0.02 ± 0.03^a^ |
| *Dokdonella* | 0.03 ± 0.03^a^ | 0.02 ± 0.01^a^ | 0.02 ± 0.03^a^ |
| *Erwinia_unclassified* | 0.04 ± 0.05^a^ | 0.48 ± 0.45^a^ | 0.02 ± 0.03^a^ |
| *Iamia* | 0.03 ± 0.01^a^ | 0.1 ± 0.07^a^ | 0.02 ± 0.03^a^ |
| *Rhodocytophaga* | 0.03 ± 0.01^a^ | 0.04 ± 0.01^a^ | 0.02 ± 0.03^a^ |
| *Rubricoccus* | 0 ± 0.01^a^ | 0.02 ± 0.01^a^ | 0.02 ± 0.03^a^ |
| *Variovorax_unclassified* | 0.04 ± 0.05^a^ | 0.04 ± 0.03^a^ | 0.02 ± 0.03^a^ |
| *Ammoniphilus_unclassified* | 0.05 ± 0^a^ | 0.04 ± 0.02^ab^ | 0.01 ± 0.02^b^ |
| *Acidovorax_unclassified* | 0.03 ± 0^a^ | 0.01 ± 0^a^ | 0.01 ± 0.02^a^ |
| *Adhaeribacter* | 0 ± 0.01^a^ | 0.01 ± 0.01^a^ | 0.01 ± 0.02^a^ |
| *Arthrospira_unclassified* | 0 ± 0^a^ | 0.01 ± 0.01^a^ | 0.01 ± 0.02^a^ |
| *Asticcacaulis_unclassified* | 0.01 ± 0.01^a^ | 0.01 ± 0^a^ | 0.01 ± 0.02^a^ |
| *Brachybacterium* | 0 ± 0^a^ | 0 ± 0^a^ | 0.01 ± 0.02^a^ |
| *Cellulosimicrobium* | 0.06 ± 0.08^a^ | 0.3 ± 0.27^a^ | 0.01 ± 0.02^a^ |
| *Flavobacterium_unclassified* | 0.07 ± 0.02^a^ | 0.05 ± 0.04^a^ | 0.01 ± 0.02^a^ |
| *Labrys* | 0.01 ± 0.01^a^ | 0.01 ± 0.01^a^ | 0.01 ± 0.02^a^ |
| *Methylotenera* | 0.03 ± 0.01^a^ | 0.01 ± 0^a^ | 0.01 ± 0.02^a^ |
| *Ochrobactrum* | 0.21 ± 0.29^a^ | 0.11 ± 0.04^a^ | 0.01 ± 0.02^a^ |
| *Prosthecobacter* | 0.03 ± 0.01^a^ | 0.05 ± 0.02^a^ | 0.01 ± 0.02^a^ |
| *Pseudonocardia_unclassified* | 0.02 ± 0.02^a^ | 0.05 ± 0.04^a^ | 0.01 ± 0.02^a^ |
| *Rhodobacter* | 0.02 ± 0.03^a^ | 0.03 ± 0.02^a^ | 0.01 ± 0.02^a^ |
| *Rhodococcus_unclassified* | 0.01 ± 0.01^a^ | 0.02 ± 0.01^a^ | 0.01 ± 0.02^a^ |
| *Virgisporangium* | 0.01 ± 0.01^a^ | 0.04 ± 0.03^a^ | 0.01 ± 0.02^a^ |
| *Pontibacter* | 0.03 ± 0^b^ | 0.05 ± 0.01^a^ | 0.01 ± 0.01^c^ |
| *Afifella* | 0.03 ± 0.01^ab^ | 0.06 ± 0.04^a^ | 0.01 ± 0.01^b^ |
| *Haliangium* | 0.01 ± 0.02^ab^ | 0.05 ± 0.03^a^ | 0.01 ± 0.01^b^ |
| *Nocardia* | 0.02 ± 0.01^ab^ | 0.04 ± 0.02^a^ | 0.01 ± 0.01^b^ |
| *Others* | 0.03 ± 0^ab^ | 0.04 ± 0.02^a^ | 0.01 ± 0.01^b^ |
| *Paenibacillus_unclassified* | 0.04 ± 0.01^a^ | 0.03 ± 0.01^ab^ | 0.01 ± 0.01^b^ |
| *Phaeospirillum* | 0.01 ± 0.02^ab^ | 0.04 ± 0.01^a^ | 0.01 ± 0.01^b^ |
| *Pimelobacter* | 0.04 ± 0.02^ab^ | 0.07 ± 0.04^a^ | 0.01 ± 0.01^b^ |
| *Solirubrobacter* | 0 ± 0^b^ | 0.02 ± 0.01^a^ | 0.01 ± 0.01^ab^ |
| *Agrobacterium_unclassified* | 0.03 ± 0.04^a^ | 0.02 ± 0.01^a^ | 0.01 ± 0.01^a^ |
| *B_42* | 0.01 ± 0.01^a^ | 0.04 ± 0.04^a^ | 0.01 ± 0.01^a^ |
| *Brevibacillus_unclassified* | 0.04 ± 0.02^a^ | 0.04 ± 0.03^a^ | 0.01 ± 0.01^a^ |
| *Candidatus_Entotheonella* | 0 ± 0.01^a^ | 0.01 ± 0^a^ | 0.01 ± 0.01^a^ |
| *Candidatus_Solibacter* | 0 ± 0^a^ | 0.01 ± 0.01^a^ | 0.01 ± 0.01^a^ |
| *Cellvibrio* | 0.01 ± 0.01^a^ | 0.03 ± 0.02^a^ | 0.01 ± 0.01^a^ |
| *Citromicrobium* | 0.04 ± 0.01^a^ | 0.03 ± 0.02^a^ | 0.01 ± 0.01^a^ |
| *Couchioplanes_unclassified* | 0 ± 0^a^ | 0.01 ± 0.01^a^ | 0.01 ± 0.01^a^ |
| *Ellin506* | 0 ± 0^a^ | 0 ± 0^a^ | 0.01 ± 0.01^a^ |
| *Gemmata* | 0.01 ± 0.01^a^ | 0.06 ± 0.04^a^ | 0.01 ± 0.01^a^ |
| *Gordonia* | 0.01 ± 0.01^a^ | 0.02 ± 0^a^ | 0.01 ± 0.01^a^ |
| *Legionella* | 0.01 ± 0.02^a^ | 0.01 ± 0^a^ | 0.01 ± 0.01^a^ |
| *Marinibacillus* | 0.01 ± 0.01^a^ | 0.01 ± 0.01^a^ | 0.01 ± 0.01^a^ |
| *Plesiocystis* | 0.02 ± 0.02^a^ | 0.02 ± 0.02^a^ | 0.01 ± 0.01^a^ |
| *Rhodococcus* | 0.02 ± 0.01^a^ | 0.02 ± 0^a^ | 0.01 ± 0.01^a^ |
| *Roseococcus* | 0 ± 0^a^ | 0.01 ± 0.01^a^ | 0.01 ± 0.01^a^ |
| *Rubellimicrobium* | 0.02 ± 0.03^a^ | 0.07 ± 0.04^a^ | 0.01 ± 0.01^a^ |
| *Salinimicrobium* | 0.01 ± 0.01^a^ | 0.01 ± 0.01^a^ | 0.01 ± 0.01^a^ |
| *Achromobacter* | 0.01 ± 0.02^ab^ | 0.05 ± 0.03^a^ | 0 ± 0^b^ |
| *Ancylobacter* | 0 ± 0^b^ | 0.01 ± 0.01^a^ | 0 ± 0^b^ |
| *Chitinophaga* | 0.01 ± 0.02^b^ | 0.06 ± 0.01^a^ | 0 ± 0^b^ |
| *Cupriavidus_unclassified* | 0 ± 0.01^ab^ | 0.01 ± 0.01^a^ | 0 ± 0^b^ |
| *Gemmatimonas* | 0 ± 0^b^ | 0.01 ± 0^a^ | 0 ± 0^b^ |
| *Georgenia* | 0.01 ± 0.01^ab^ | 0.05 ± 0.03^a^ | 0 ± 0^b^ |
| *Nannocystis* | 0 ± 0.01^b^ | 0.02 ± 0^ab^ | 0 ± 0^b^ |
| *Nannocystis_unclassified* | 0.01 ± 0.01^ab^ | 0.01 ± 0^a^ | 0 ± 0^b^ |
| *Nitrospira* | 0.01 ± 0.01^ab^ | 0.01 ± 0.01^a^ | 0 ± 0^b^ |
| *Rhodoplanes_unclassified* | 0 ± 0^b^ | 0.01 ± 0^a^ | 0 ± 0^b^ |
| *Sanguibacter* | 0 ± 0^ab^ | 0 ± 0^a^ | 0 ± 0^b^ |
| *Actinomycetospora* | 0 ± 0^a^ | 0.03 ± 0.03^a^ | 0 ± 0^a^ |
| *Aquicella* | 0.01 ± 0.01^a^ | 0.01 ± 0.01^a^ | 0 ± 0^a^ |
| *Arthrobacter* | 0 ± 0^a^ | 0.01 ± 0.01^a^ | 0 ± 0^a^ |
| *Candidatus_Protochlamydia* | 0 ± 0^a^ | 0.01 ± 0.01^a^ | 0 ± 0^a^ |
| *Cellulomonas* | 0 ± 0^a^ | 0.01 ± 0.01^a^ | 0 ± 0^a^ |
| *Cellulomonas_unclassified* | 0.01 ± 0.01^a^ | 0.06 ± 0.06^a^ | 0 ± 0^a^ |
| *Chloronema* | 0.02 ± 0.03^a^ | 0.06 ± 0.06^a^ | 0 ± 0^a^ |
| *Euzebya* | 0 ± 0^a^ | 0.01 ± 0.01^a^ | 0 ± 0^a^ |
| *Flavihumibacter* | 0.02 ± 0.03^a^ | 0.04 ± 0.03^a^ | 0 ± 0^a^ |
| *Glycomyces* | 0.05 ± 0.07^a^ | 0.05 ± 0.06^a^ | 0 ± 0^a^ |
| *Janthinobacterium* | 0.01 ± 0.01^a^ | 0.01 ± 0^a^ | 0 ± 0^a^ |
| *Kushneria* | 0 ± 0^a^ | 0.01 ± 0.02^a^ | 0 ± 0^a^ |
| *Myxococcus* | 0 ± 0^a^ | 0.02 ± 0.02^a^ | 0 ± 0^a^ |
| *Olivibacter* | 0.06 ± 0.08^a^ | 0.09 ± 0.13^a^ | 0 ± 0^a^ |
| *OR_59* | 0 ± 0^a^ | 0 ± 0^a^ | 0 ± 0^a^ |
| *Pleomorphomonas* | 0 ± 0^a^ | 0.02 ± 0.02^a^ | 0 ± 0^a^ |
| *Pseudomonas* | 0.03 ± 0.04^a^ | 0.11 ± 0.14^a^ | 0 ± 0^a^ |
| *Rickettsia* | 0.01 ± 0.02^a^ | 0.01 ± 0.01^a^ | 0 ± 0^a^ |
| *Rubrobacter* | 0 ± 0^a^ | 0.01 ± 0.01^a^ | 0 ± 0^a^ |
| *Shinella_unclassified* | 0 ± 0.01^a^ | 0.01 ± 0^a^ | 0 ± 0^a^ |
| *Sorangium* | 0.01 ± 0.01^a^ | 0.02 ± 0.02^a^ | 0 ± 0^a^ |
| *Sphingobium_unclassified* | 0 ± 0^a^ | 0.03 ± 0.06^a^ | 0 ± 0^a^ |
| *Sporichthya* | 0 ± 0^a^ | 0.01 ± 0.01^a^ | 0 ± 0^a^ |
| *Virgibacillus* | 0.02 ± 0.01^a^ | 0.02 ± 0.02^a^ | 0 ± 0^a^ |
| *Xylanimicrobium_unclassified* | 0.01 ± 0.01^a^ | 0.02 ± 0.03^a^ | 0 ± 0^a^ |
